# High-throughput profiling of point mutations across the HIV-1 genome

**DOI:** 10.1186/s12977-014-0124-6

**Published:** 2014-12-19

**Authors:** Laith Q Al-Mawsawi, Nicholas C Wu, C Anders Olson, Vivian Cai Shi, Hangfei Qi, Xiaojuan Zheng, Ting-Ting Wu, Ren Sun

**Affiliations:** Department of Molecular and Medical Pharmacology, David Geffen School of Medicine, University of California, Los Angeles, CA 90095 USA; AIDS Institute, University of California, Los Angeles, CA 90095 USA; Molecular Biology Institute, University of California, Los Angeles, CA 90095 USA

**Keywords:** HIV-1, Next-generation sequencing, Mutagenesis

## Abstract

**Background:**

The HIV-1 pandemic is not the result of a static pathogen but a large genetically diverse and dynamic viral population. The virus is characterized by a highly mutable genome rendering efforts to design a universal vaccine a significant challenge and drives the emergence of drug resistant variants upon antiviral pressure. Gaining a comprehensive understanding of the mutational tolerance of each HIV-1 genomic position is therefore of critical importance.

**Results:**

Here we combine high-density mutagenesis with the power of next-generation sequencing to gauge the replication capacity and therefore mutational tolerability of single point mutations across the entire HIV-1 genome. We were able to achieve the evaluation of point mutational effects on viral replicative capacity for 5,553 individual HIV-1 nucleotide positions – representing 57% of the viral genome. Replicative capacity was assessed at 3,943 nucleotide positions for a single alternate base change, 1,459 nucleotide positions for two alternate base changes, and 151 nucleotide positions for all three possible alternate base changes. This resulted in the study of how a total of 7,314 individual point mutations impact HIV-1 replication on a single experimental platform. We further utilize the dataset for a focused structural analysis on a capsid inhibitor binding pocket.

**Conclusion:**

The approach presented here can be applied to any pathogen that can be genetically manipulated in a laboratory setting. Furthermore, the methodology can be utilized under externally applied selection conditions, such as drug or immune pressure, to identify genetic elements that contribute to drug or host interactions, and therefore mutational routes of pathogen resistance and escape.

**Electronic supplementary material:**

The online version of this article (doi:10.1186/s12977-014-0124-6) contains supplementary material, which is available to authorized users.

## Background

Currently ~35 million people are living with human immunodeficiency virus-1 (HIV-1) infection, the pathogen responsible for acquired immunodeficiency syndrome (AIDS), with tens of millions having died of AIDS-related causes worldwide since the pandemic began (UNAIDS. GAP Report; 2013). The virus rapidly evolves due to the high error rate of the viral reverse transcriptase (RT) enzyme at 3.4 × 10^−5^ mutations per site per generation coupled with a rapid generation output rate of ~1 × 10^10^ virions per patient per day [1-4], and the propensity of RT to mediate RNA recombination via template switching during genomic reverse transcription at ~10 times per replication cycle [5,6]. This genetic plasticity renders many vaccine candidates effective at neutralizing only a subspecies of the virus within the patient, and drives the ongoing challenge of antiretroviral resistance in HIV-1 therapy. It is therefore of paramount importance that we gain an understanding of the mutational tolerance of the HIV-1 genome in exquisite detail to effectively design strategies to prevent, treat, and ultimately diminish the damage to human health. Here we provide a replication capacity (RC) analysis of 57% of the HIV-1 genome for single point mutations using a high-throughput genetic approach that combines high-density mutagenesis with the power of next-generation sequencing (NGS) we term quantitative high resolution genetics (qHRG). The RC dataset can be further used to assist in HIV-1 vaccine design, identification of nucleotide-level cis functionalities, and structural annotation to aid in drug development - an example of which, using the viral capsid protein, is given. qHRG can be applied to other viral pathogens and under any applied selection condition that may be relevant to viral pathogenesis.

## Results

### Point mutation library construction

**Figure 1:**
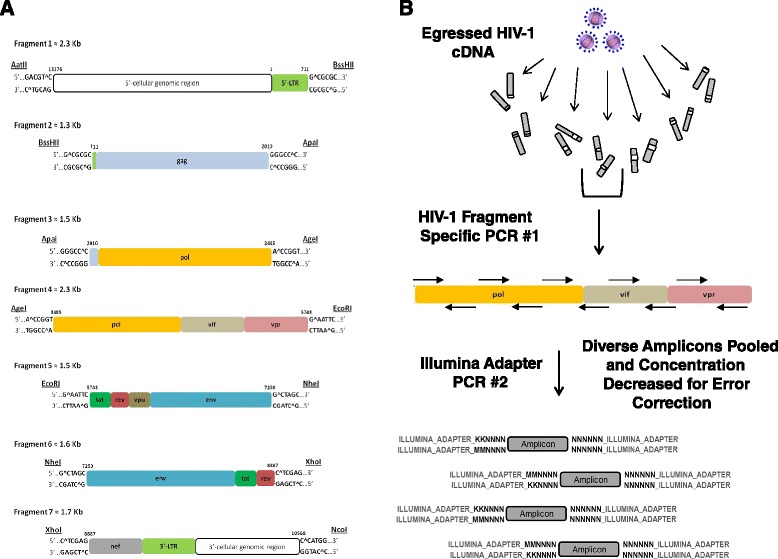
**HIV-1 mutant library and NGS sample preparation. (A)** Genomic region for each HIV-1 mutation library. **(B)** The two-step PCR amplicon approach for NGS sample preparation. Virion cDNA from each mutant viral population cell passage is used as template for a HIV-1 specific staggered PCR step that uses primers specific to the HIV-1 mutagenized region containing overhangs with a complex 10 ‘N’ nucleotide tag with two keto “K” or amino “M” nucleotide positions that identify the specific fragment and population, respectively. PCR products from step one are pooled, the amplicon molecule concentration is accurately measured, and then decreased for error correction. The pooled sample is then used as template for a second PCR using a single primer set containing the remainder of the Illumina adapter region for NGS.

To demonstrate the utility of qHRG on an entire viral genome we aimed to generate highly dense mutagenic HIV-1 genomic libraries and monitor the variant frequency change in the population following cell passage. Each HIV-1 library was generated using the pNL4-3 proviral plasmid as template for PCR with an error-prone polymerase. Primer pairs corresponding to eight unique restriction sites that segmented the genome into seven fragments ranging in size from ~1.3 kb to 2.3 kb (Figure 1A) were used. Mutant PCR fragments were subcloned back into pNL4-3, creating seven distinct mutant libraries, where one of the seven fragments contained point mutations, and the remaining six were genetically wild type (WT). Our fragment based design of seven distinct libraries was used to maximize sequencing of only targeted mutational fragments on the finite Illumina NGS platform and not waste resources sequencing WT genomic regions not targeted for error-prone PCR. Unique molecules obtained after subcloning were maximized to ensure a comprehensive point mutational analysis in each distinct HIV-1 proviral fragment. We generated an average of five mutations per genomic kilobase in each library, an amount chosen to be high enough to detect mutation frequency change above the de novo rate and limit sequencing demand. We additionally aimed to achieve significant coverage at each nucleotide point mutation to minimize the possible epistatic effects other mutations associated with the position under examination would have on our RC calculations. We hypothesized this approach would ‘average out’ possible epistatic effects between the multiple mutations inherent in our experimental design (Additional file 1). To completely eliminate the possibility of any engineered epistatic influence on replication, however, is to create a library containing only a single mutation per genome – a rate which becomes more practical to assess point mutational effects on RC in high throughput with advances in NGS technology. The libraries obtained were highly complex, consisting ~ 40,000 to 130,000 unique viral genomes, with each nucleotide position covered 77 to 204-fold. The total number of distinct mutant HIV-1 genomes is approximately half a million.

### Mutant library selection and next-generation sequencing

Each mutant plasmid library was reconstituted into virus, and passaged in CEM T-lymphocytes in an iterative and parallel fashion for four rounds. For each passage, supernatant was collected, titered, and added to ~20 million cells at a MOI of 0.01 to initiate the next selection round. Virion RNA was isolated after each passage, reversed transcribed, and quantified using qPCR. Transcript levels for each input plasmid and all selection rounds were high enough to maintain the full complexity of each starting library, and we therefore could accurately quantify relative frequencies of each variant following each round. Initial deep sequencing experiments on single amplicon stretches within the gag, pol, and env genes of the viral genome for each passage round indicated RC selection was largely complete by passage round 2 (R2). Furthermore, mutant complexity is largely retained from DNA plasmid to reconstituted viral particles during the transfection step, with a correlation of 85-95% across different libraries. We therefore focused on the input plasmid libraries and R2 egressed viral libraries for RC analysis across the entire HIV-1 genome (qPCR transcript levels for each shown in Additional file 2A). The complete cell culture passage scheme is provided in Additional file 3. We conceptually designed a two-step PCR strategy to prepare isolated virion cDNA that is specific for the NGS Illumina platform (Figure 1B). Following HIV-1 cDNA generation, the first PCR step utilizes HIV-1 library specific primers to generate short (~188 bp) amplicons with each containing a unique nucleotide sequence tag and constant regions at each terminus corresponding to the adaptor regions required for the Illumina sequencing platform. The generation of each PCR product is confirmed by electrophoresis, and library-specific amplicons are pooled and subjected to a single sub-saturation PCR affixing the remaining Illumina adaptor region required for NGS. The final products were then sequenced on an Illumina HiSeq2000 machine using paired-end 2 × 100 read parameters. The amplicon-based technique ensures uniform representation of the entire genome as compared to traditional shearing methods which often result in over-representation of DNA fragment ends. The approach also affords unique nucleotide sequence tags and the adapter region specific for the Illumina platform in the primer design, and includes an error-correction step to clearly distinguish true mutation versus NGS instrument error from the output sequencing reads. Our strategy ensures an accurate count for each discrete amplicon present in the selection pool, which through clustering of unique sequence tags present on each amplicon, quickly identifies sequencing errors, a procedure conceptually similar to a method previously described [7]. However, our strict limitation on redundancy ensures sequence space is maximized to achieve error correction without losing sequencing depth on the finite Illumina platform. Another key for confident quantification of relative frequencies in this strategy is to have a diverse enough combination of unique sequence tags to cover all individual WT and mutant species present in the selection pool (see Methods).

### Calculation of point mutation replication capacity

**Figure 2:**
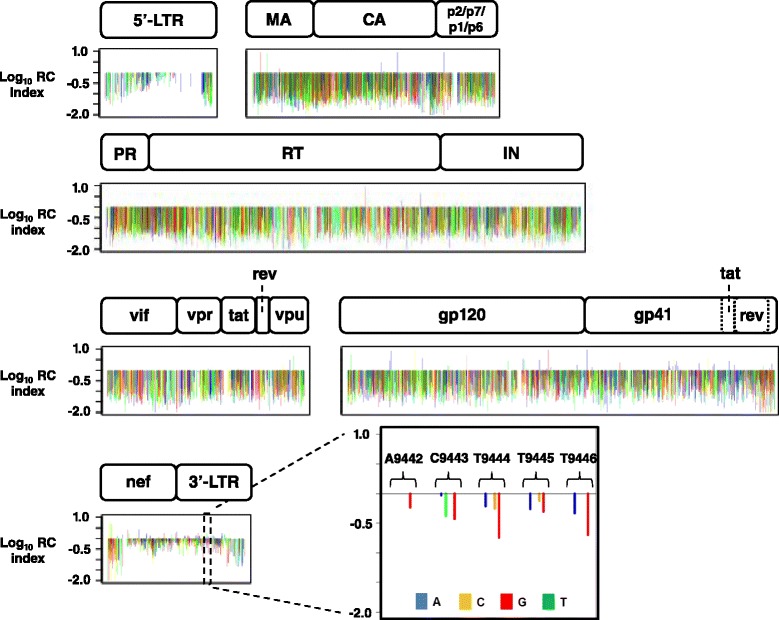
**RC map for 57% of nucleotide positions in the pNL4-3 HIV-1 genome.** The HIV-1 genome showing the log_10_ RC index for all 7,314 individual point mutations assessed for RC after library passage in CEM T-lymphocyte cell culture. HIV-1 genomic regions are identified. Color denotes nucleotide identity of base change: A: blue, C: yellow, G: red, and T: green. An inset high-resolution view of the 3’-LTR region (9442–9446) of the genome is also highlighted.

The complete RC map of the HIV-1 genome is depicted in Figure 2. All mutations with a low number of input sequence counts (<30) were filter removed from analysis. We obtained RC data at single nucleotide resolution for 57% of the viral genome. Our target sensitivity was to detect variants as rare as 0.001% of the population, and achieved this for the majority of amplicons. However, sensitivity was dependent on amplicon coverage per selection round, and generally varied from 0.0004-0.009% (Additional file 2B and Additional file 4). Overlapping amplicon regions where counts and sensitivity were increased is also listed in Additional file 4. The presence of overlapping amplicon regions, produced from separate PCR primers, also allowed us to validate the reproducibility of our method and confirm the variability of PCR efficiencies when using differently tagged primers was minimal. We analyzed the occurrence frequency and RC data of each individual mutant present on all overlapping amplicons. As seen in Additional file 5, the data shows a high correlation (~90%) for all overlapping amplicons (arbitrarily termed ‘amplicon 1’ and ‘amplicon 2’ in graphs) for the DNA input library and R2. This data indicates a very reproducible platform to assess mutational impact. Quantitative comparison of the genetic compositions of each mutant library pre- (input plasmid) and post-selection (R2) provides indication of the relative RC for each individual viral mutation, and is expressed as a RC index:

RC index = (occurrence frequency in R2)/(occurrence frequency in plasmid library).

**Figure 3:**
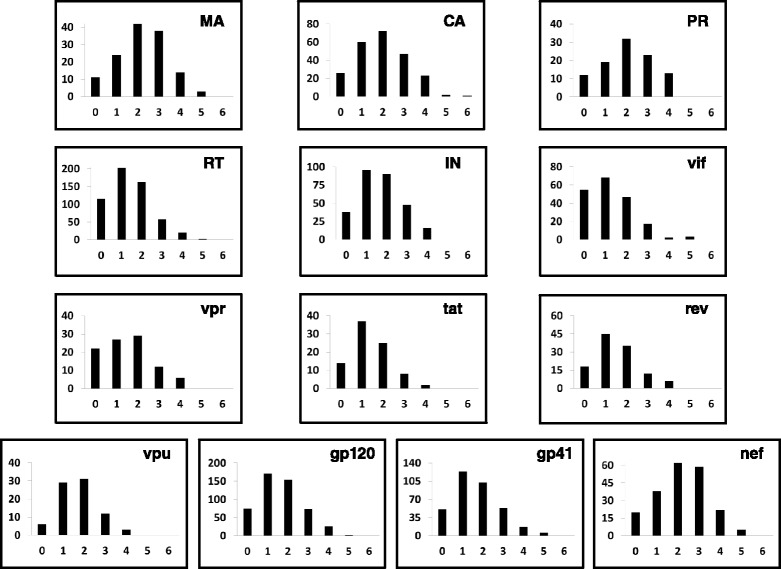
**Missense mutational profile coverage at the HIV-1 amino acid level.** The number of missense substitutions (x-axis) quantified for RC at each HIV-1 codon (y-axis) is grouped by HIV-1 protein and provided as separate histograms. Our profile predominately achieved coverage of 1–3 missense substitutions at each interrogated codon for all viral proteins.

In total, 5,553 individual HIV-1 nucleotide positions were interrogated by mutations (57% of the genome). HIV-1 RC was assessed at 3,943 nucleotide positions for a single alternate base change, 1,459 nucleotide positions for two alternate base changes, and 151 nucleotide positions for all three possible alternate base changes, resulting in a total assessment of 7,314 individual point mutations. Based on a survey of HIV-1 pNL4-3 mutational phenotypes described in the literature we determined that the approximate cutoff points for lethal, attenuated, and tolerated mutational RC index values are ≤0.1, from 0.1 – 0.2, and ≥0.2, respectively. It should be noted that the numerical scale presented in our mutational RC dataset should be treated as a gradient scale as opposed to fixed finite numerical values. Therefore, caution should be exercised when deducting definitive phenotypic conclusions for mutational RC index values measured at our approximate cut-off points. In general, 51% of the point mutations analyzed were lethal, 20% were attenuated, and 29% displayed a tolerated phenotype. Depicted in Figure 2 is the log_10_ RC index for all 7,314 individual HIV-1 point mutations, with an inset view of the 3’ LTR region of the genome highlighting the detailed information garnered from the qHRG platform. A nucleotide stretch from position 9442–9446 is shown in high resolution depicting the RC impact of mutations by nucleotide identity. The complete dataset is deposited to the NCBI Sequence Read Archive (SRA) under accession code BioProject PRJNA259391. The RC index values for all HIV-1 missense substitutions and mutations in non-coding regions are provided in Additional file 6. Many silent mutations in the gag and enzymatic pol region have lower RC index values than expected. Although this may indicate functionality at the nucleotide level, it is also possible that our mutation rate (~5 mutations per kb) resulted in an amplified ‘dragging-down’ effect for neutral mutations especially in enzymatic protein coding regions, where subsequent deleterious mutations present on the genome with the mutation of interest created an average RC value to be lower than expected. We therefore focused the remainder of our analysis on the missense mutational dataset. Profile coverage at the amino acid level is shown by HIV-1 protein in Figure 3 with the number of missense substitutions (x-axis) for each amino acid position (y-axis) measured for RC. Our profile predominately achieved 1 to 3 missense substitutions at each interrogated codon with substitution identities dependent on what can be obtained by a single base change due to the error-prone mutagenic PCR strategy used for library construction.

### Validation of missense mutation replication capacity

**Figure 4:**
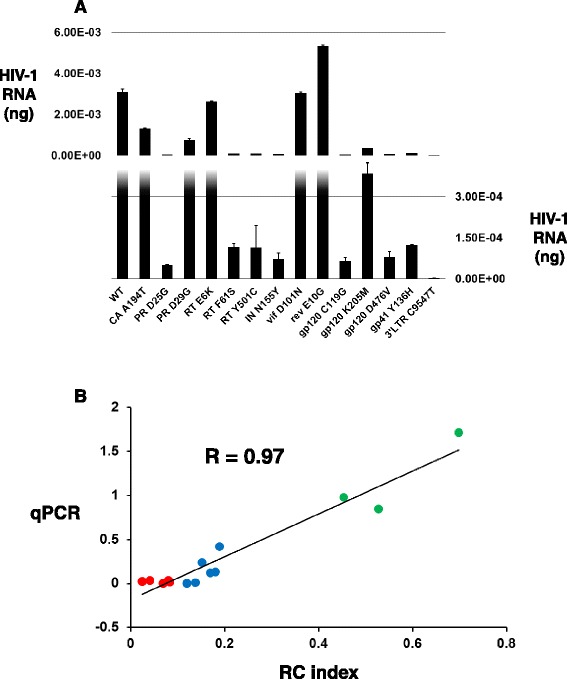
**Validation of the HIV-1 qHRG missense mutational profile. (A)** Virion RNA amounts in nanogram for WT and each viral mutant following CEM T-lymphocyte infection quantified by qPCR. Split histogram shown with average amount of ng RNA from two separate replicates provided on vertical right axis for lower panel, and vertical left axis for upper panel. HIV-1 mutant identity is given on x-axis. **(B)** Correlation between the profile RC index (x-axis) and the qPCR results [ng mutant RNA]/[ng WT RNA] (y-axis) of individual HIV-1 mutants showing a very high Pearson’s correlation of R = 0.97. Data points are colored according to profile phenotype: tolerated: green, attenuated: blue, and lethal: red.

We randomly selected a set of 13 different missense substitutions that covered a range of RC phenotypes throughout the HIV-1 genome for validation. We included the replicative incompetent protease active site substitution D25G as a negative control, and compared all mutant virion RNA levels to WT. Information pertaining to the HIV-1 genomic region, amino acid substitution, corresponding DNA mutation, initial DNA coverage, and a comparison of the RC index and qPCR validation results for each substitution are shown in Table 1. All mutant viruses were constructed individually in pNL4-3, sequence validated, and used to infect CEM T-lymphocyte cells at a MOI of 0.01 under the same experimental conditions used for the qHRG profile. Virion RNA was reversed transcribed, and quantified using qPCR. The nanogram RNA amount for WT and each viral mutant is shown in Figure 4A. Shown in Figure 4B is the correlation between the RC index and the qPCR results, the latter expressed as the fraction [ng mutant RNA]/[ng WT RNA]. The RC index and qPCR results were very well correlated, with a Pearson’s correlation of R = 0.97, indicating the results of the profile largely predict how substitutions will impact HIV-1 replication. We also compared the RC index results to the literature, focusing on substitutions reported in the integrase region of the genome. We observed an 80% correlation between our profiling dataset and twenty different integrase viral variants compiled from eleven separate manuscripts when comparing lethal versus non-lethal replicative phenotypes (Additional file 7 – references included).

**Table 1:** **qHRG missense mutation experimental validation**

**HIV-1 genomic region**	**Substitution**	**DNA mutation**	**DNA coverage** ^**a**^	**RC index**	**qPCR value** ^**b**^
Capsid	A194T	G1765A	216	0.189	0.421
Protease	D25G	A2326G	165	0.068	0.002^c^
Protease	D29G	A2338G	178	0.151	0.241
Reverse transcriptase	E6K	G2565A	353	0.527	0.849
Reverse transcriptase	F61S	T2731C	150	0.079	0.037
Reverse transcriptase	Y501C	A4051G	190	0.04	0.037
Integrase	N155Y	A4692T	90	0.119	0.002^c^
vif	D101N	G5341A	223	0.453	0.981
rev	E10G	A5997G	292	0.698	1.714
gp120	C119G	T6575G	123	0.082	0.021
gp120	K205M	A6834T	337	0.169	0.123
gp120	D476V	A7635T	383	0.024	0.026
gp40	Y136H	T8126C	206	0.137	0.012^c^
3’-LTR	N/A	C9547T	234	0.18	0.132^c^

^a^Number of mutation initial sequence counts.

^b^Value obtained from [ng RNA mutant]/[ng RNA WT].

^c^For mutants where [ng RNA] < 0.01, ELISA measured [ng p24 mutant]/[ng p24 WT] ratios were averaged into value reported.

### Application of missense mutation replication capacity profile

Structural annotation of qHRG RC data can provide valuable insight to help explain functionalities of viral proteins for a multitude of aspects relevant to viral replication and disease progression. Protein RC views in three-dimensional space allow for the detection of structurally adjacent positions with similar RC costs upon substitution not readily apparent if focusing on a specific protein region on the primary sequence. This can aid in assessing the genetic barriers of resistance in existing drug binding sites to guide inhibitor optimization, and can provide for the discovery of altogether novel binding pockets, where regions of low substitution tolerability can be used for therapeutic development in combination with computational techniques.

**Figure 5:**
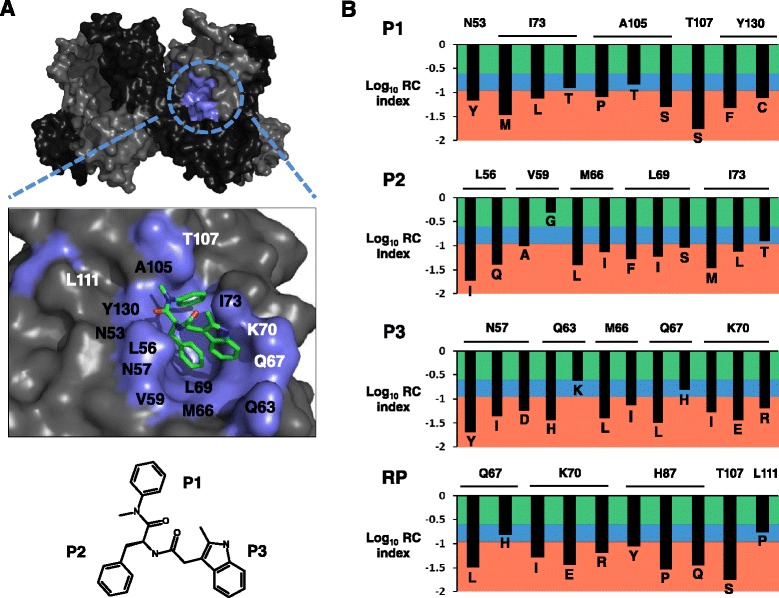
**Application of the HIV-1 qHRG missense mutational profiling data to aid in CA drug design. (A)** Surface representation of CA hexamer (PDB 3H47), with alternating CA monomers shown in light and dark grey, respectively. The CA binding pocket of PF-3450074 is colored in cyan. High-resolution inset view also provided highlighting inhibitor binding orientation (PDB 2XDE), with CA protein in light grey, inhibitor binding residues in cyan, and PF-3450074 in stick representation and colored by element with C in green, O in red, and N in blue. CA positions are labeled to show drug binding residues (black) and/or resistant positions (white). The small molecule chemical structure of PF-3450074 is provided with chemical moieties that occupy each CA sub-pocket labeled. **(B)** The log_10_ RC index for CA substitutions at positions involved in PF-3450074 binding (P1-P3) and the resistant positions (RP). The graphs are color-coded to represent a tolerant (green), attenuated (blue), or lethal (red) phenotype depending on the measured log_10_ RC index for each CA substitution. WT amino acid identities are horizontally labeled at the top of each graph, with substitution identities individually labeled for each entry.

We focused our HIV-1 missense RC dataset analysis on the capsid (CA) protein, which is essential for replication and is originally synthesized as the central component of the structural 55 kDa gag polyprotein. CA mediates immature virion assembly as well as forms a conical shell comprised of ~1500 molecules as a predominately hexameric lattice to enclose the viral RNA diploid genome and associated HIV-1 enzymes upon gag cleavage and viral maturation [8-11], and lattice disassembly is an obligatory step early in the replication cycle [12]. Targeted disruption of the CA conical assembly therefore represents an important unexploited target for the development of novel HIV/AIDS therapeutics [13-15]. Recently, a newly discovered CA inhibitor (PF-3450074) displaying an antiviral EC_50_ of 0.57 ± 0.26 μM with a favorable therapeutic index of 121 was reported in the literature [16,17]. The inhibitor binding region on the hexameric CA assembly (PDB 3H47) [18], with a labeled inset view of the co-crystal CA-inhibitor binding orientation (PDB 2XDE) [16], and the chemical structure of PF-3450074 is shown in Figure 5A. PF-3450074 specifically binds at the mature CA-CA protein interface, makes contacts within three binding sub-pockets (P1-P3), and destabilizes the higher-ordered capsid cone resulting in potent antiviral activity [16]. HIV-1 viral passage experiments using PF-3450074 chemical analog selection pressure revealed the primary resistant substitution T107N with secondary substitutions Q67H, K70R, H87P, and L111I after 53 days [16]. Interestingly, mutant virus containing the individual T107N substitution or all five substitutions were reported to replicate comparably to WT in reporter gene-based infection assays [16]. However, previous studies have revealed the importance of these positions for CA function and/or viral replication [19-22]. Our qHRG data, strictly measuring RC in cell culture, also reveals a predominately high RC cost for substitutions arising in the binding pocket of PF-3450074 and for the resistance positions identified, indicating a very favorable drug binding site for the design of CA targeted antivirals that may experience prolonged effectiveness upon clinical use. Shown in Figure 5B is the log_10_ RC index for CA substitutions quantified in our profile for each residue involved in PF-3450074 binding (P1-P3) and the resistant positions (RP) observed after serial viral passage under inhibitor pressure. The graphs are color-coded to represent a tolerant (green), attenuated (blue), or lethal (red) phenotype depending on the measured log_10_ RC index for each CA substitution. For P1, CA substitutions N53Y, I73M/L, A105P/T/S, T107S, and Y130F/C all displayed a lethal RC. Two CA substitutions in P1, I73T and A105T, both attenuated with a RC index of 0.12 and 0.14, respectively, were the only replicatively viable substitutions. At CA P2, L56I/Q, V59A, M66L/I, L69F/I/S, and I73M/L were all lethal for replication, with V59G displaying a tolerated phenotype (0.48), and I73T attenuated as in P1. At CA P3, N57Y/I/D, Q63H, M66L/I, Q67L, and K70I/E/R all displayed a lethal RC, with Q63K tolerated for RC (0.24), and Q67H displaying an attenuated phenotype (0.15). At the CA positions identified as conferring PF-3450074 resistance upon substitution, only the attenuated positions Q67H, located in P3, and L111P (0.17) were viable for viral replication. All other CA RP substitutions, Q67L, K70I/E/R, H87Y/P/Q, and T107S, were lethal for RC. The comprehensive RC information provided here can further aid to maximize the redesign of next-generation inhibitors binding this CA region while avoiding amino acid substitution routes with high RC that would likely lead to the rapid emergence of viral resistance upon inhibitor pressure. The use of RC profiling data to inform rational in silico drug design approaches helps to refine next-generation antiviral inhibitor design by providing the substitution routes likely to result in the emergence of inhibitor resistant viral variants.

## Discussion

In this study we used our high-throughput genetic platform to assess the impact of point mutations on viral RC in an X4 laboratory HIV-1 strain in T-lymphocyte cell culture. The RC of mutations at each nucleotide position strictly reflects this experimental condition. Interestingly, mutations that resulted in a RC greater than the WT pNL4-3 strain (RC index >1) were very rare and amounted to only 1.9% of the complete dataset (140 out of 7,314 total mutations) providing indication that pNL4-3 is already well adapted to cell culture growth and optimization resulting from substitutions, at least resulting from one mutational step away from WT, is not a common occurrence. Our methodology could be utilized to define nucleotide positions that confer a RC advantage under other selection conditions such as HLA escape or drug resistance, or other genetic backbones such as a M-tropic CCR5 utilizing strain. Employing qHRG under different HIV-1 relevant selection conditions with multiple genotypes and compiling the data will provide a more comprehensive map of the genetics of HIV-1 pathology. Moreover our RC data may overlap with sequence conservation information that is readily available from patient derived HIV-1 sequence databases, but conservation of a particular nucleotide position does not strictly equate to a high RC for that viral position [23,24], but a direct RC advantage of a select ancestral strain in that particular patient environment. qHRG provides a complementary, direct, and functionally-based approach to impartially identify amino acid residues that are critical for viral replication in a defined cellular environment. To complement data collected from naturally occurring variations in clinical samples, our approach can be applied to study the dynamics of viral mutant populations in different growth conditions with precise control of experimental conditions to directly ascertain the mechanistic interplay between virus and host.

In the course of applying our genetic profiling approach on the complete HIV-1 genome, we have identified a number of improvements that can be incorporated in the future usage of our methodology. Since multiple mutations were introduced for individual clones in the mutant libraries in this study, a systematic mutational additive effect may be present during the quantification of individual mutation RC. It is possible that our mutation rate (~5 mutations per kb) resulted in a ‘dragging-down’ effect for neutral mutations especially in enzymatic protein coding regions, where subsequent deleterious mutations present on the genome with the mutation of interest created an average RC value to be lower than expected. The obtainment of larger mutant library pools, which would afford a higher coverage at each nucleotide position, would considerably improve the RC data at silent mutational positions exhibiting a neutral phenotype. Longer sequencing reads, which are becoming increasingly available on multiple NGS platforms, would also address epistatic effects. Additionally, this problem can be resolved by lowering the number of mutation rate to 1–2 per clone, which becomes more feasible and affordable with the increasing capacity of NGS technology. In addition, the high mutation rate within the HIV-1 replication cycle may increase the noise of RC profiling and obscure the identification of a lethal mutation. Mutational assessment could be further refined by increasing the input occurrence frequency of individual point mutations by performing random mutagenesis on a shorter fragment. A more dramatic drop of occurrence frequency will be detected if a lethal mutation has a higher occurrence in the input mutant library. In other words, the calculated RC value will be lower for a lethal mutation that has a higher occurrence in the input mutant library. We anticipate the above technical improvements would enrich the quality of the RC profiling data.

This study represents the first application of our qHRG method to an entire viral genome – namely HIV-1. However, our platform will be useful for any virus that can be genetically manipulated in a laboratory setting. We recently demonstrated the power of our sequencing approach for viral drug development using the NS5A protein of hepatitis C (HCV) under the inhibitory pressure of Daclatasvir to help predict clinical outcomes if development continued to therapeutic use [25]. For influenza A, we have profiled RC of the hemagglutinin gene at single-nucleotide resolution [26], profiled for mutations affecting type 1 interferon sensitivity in the NS segment [27], and uncovered compensatory mutations to Tamiflu in the neuraminidase gene [28]. As demonstrated in our HCV study, we show how the comprehensiveness of qHRG can be increased by applying saturation mutagenesis for library construction, which enables the interrogation of every codon for all possible amino acid substitutions, and removes the experimental limitation of only examining substitutions that can be obtained by one mutational step away from WT. We have also demonstrated the application of our amplicon-based PCR approach for Illumina NGS to clinical HIV-1 quasi-species populations in acute infection [29] and achieved a higher sensitivity in identifying rare quasi-species variants as compared to published approaches using other NGS platforms. A number of groups have also been utilizing high-resolution mutational scanning combined with NGS that targets proteins or protein domains to gain insight into protein function and evolutionary mutational tolerance [30-35].

## Conclusions

We have provided a RC map of the HIV-1 genome using a genetic platform that combines high-density mutagenesis with NGS. The utility of such a comprehensive RC dataset is extensive. Examples include (i) determining regions less tolerable to mutation to aid in vaccine or therapeutic development, (ii) identification of nucleotide sequence changes that result in a lethal replication phenotype, but encode silent substitutions at the amino acid level – suggesting function at the nucleic acid level, ie., RNA secondary structure, DNA-protein recognition signals, or small RNAs, and (iii) structural annotation of essential amino acids on existing three-dimensional structures to provide insight into structure-function relationships. Here, the power of our qHRG platform is the ability to sensitively quantify the RC of individual viral variants in a large and diverse population of mutants for involvement in a replicative pathogenic process within a well-defined biological environment on a single experimental platform.

## Methods

### Viral mutant library preparation

To generate the HIV-1 mutant library we designed a PCR strategy utilizing the HIV-1 proviral DNA plasmid pNL4-3 as template and the error-prone polymerase Mutazyme II (Strategene) to generate the point mutations during PCR amplification. The HIV-1 genome of the molecular clone pNL4-3 was divided into 7 segments ranging from ~1.3 to 2.3 Kb. Fragment start and end sites were selected based on the location of unique enzyme digest restriction sites within the plasmid. We further designed primer sets overlapping each distinct restriction site for error-prone PCR and validated the primer pairs for efficient PCR amplification. All primers used in this study are given in Additional file 8. Error-prone PCR was conducted using the GeneMorph II Random Mutagenesis Kit (Stratagene) and a starting target mutagenized fragment region amount of either 0.5 ng (fragment 1, 2, and 6) or 5 ng (fragment 3, 4, 5, and 7). All error-prone PCR reactions contained an initial melt step at 95°C for 2 min and a final extension step at 72°C for 10 min, followed by a final hold at 4°C. Repetitive error-prone PCR cycle parameters were fragment specific according to optimized primer annealing temperatures, extension times due to fragment length, and cycle numbers to obtain a target mutation rate of as close to ~5 mutations per kilobase as possible. For each fragment: Frag1: 95°C 30 sec, 63°C 30 sec, 72°C 2 min 15 sec, 40 cycles; Frag2: 95°C 30 sec, 55°C 30 sec, 72°C 1 min 30 sec, 30 cycles; Frag3: 95°C 30 sec, 63°C 30 sec, 72°C 1 min 30 sec, 30 cycles; Frag4: 95°C 30 sec, 63°C 30 sec, 72°C 2 min 15 sec, 40 cycles; Frag5: 95°C 30 sec, 55°C 30 sec, 72°C 1 min 30 sec, 30 cycles; Frag6: 95°C 30 sec, 56°C 30 sec, 72°C 1 min 30 sec, 30 cycles; Frag7: 95°C 30 sec, 65°C 30 sec, 72°C 1 min 30 sec, 40 cycles. To eliminate WT background contamination we constructed seven new pNL4-3 vectors, where we swapped each ~1.3-2.3 kb WT fragment with a small 15 nucleotide fragment that contained the corresponding restriction sites at either end and a new unique MluI site within the fragment as a “kill-site” not originally present in the pNL4-3 vector. The seven vectors were strictly used to sub-clone each mutant fragment PCR product back into pNL4-3 resulting in a full length proviral genome, and enabled us to use PCR clean-up columns to remove the 15 base pair insert after vector digestion resulting in very clean ligations. This cloning strategy further ensured no WT background species will contaminate our libraries by (1) using the MluI kill-site to further remove background, and (2) guaranteed that if background was present after ligation the viral genomes would be missing greater than a kilobase of genome and result in non-viable viral particles. Mutagenized PCR fragments were ligated into each corresponding digested cloning vector using T4 DNA ligase (Invitrogen), transformed into chemically competent DH5α (fragments 2 and 3) or electroporated using a Gene Pulser II (BioRad) into MegaX DH10B T1R (Invitrogen) E.Coli according to manufacturer’s instructions and plated on four 143 cm^2^ ampicillin agar plates. For each fragment, colonies were counted, scraped and pooled into ~25 mL LB and the plasmid was midi-prepped (Invitrogen). The mutation rate per fragment and coverage for each fragment nucleotide position base change are as follows: Frag1: 4.5 mutations per 711 base pair region; 64,781 colonies obtained resulting in 134-fold coverage for all mutations at each fragment position. Frag2: 6 mutations per ~1300 base pair region; 49,968 colonies obtained resulting in 77-fold coverage for all mutations at each fragment position. Frag3: 10 mutations per ~1500 base pair region; 39,240 colonies obtained resulting in 87-fold coverage for all mutations at each fragment position. Frag4: 14 mutations per ~2300 base pair region; 54,605 colonies obtained resulting in 110-fold coverage for all mutations at each fragment position. Frag5: 8.5 mutations per ~1500 base pair region; 62,598 colonies obtained resulting in 118-fold coverage for all mutations at each fragment position. Frag6: 6 mutations per ~1600 base pair region; 79,750 colonies obtained resulting in 99.5-fold coverage for all mutations at each fragment position. Frag7: 4 mutations per 832 base pair region; 127,406 colonies obtained resulting in 204-fold coverage for all mutations at each fragment position.

### Passage of HIV-1 mutant libraries in CEM T-lymphocyte cell culture

Each HIV-1 mutant plasmid library was separately transfected in 293T cells for viral propagation. Cell culture supernatant after transfection and after each CEM T-cell passage was measured for p24 levels using the CFAR Virology Core Facility at UCLA, filtered with a 0.22 μM MCE filter (Fisher Scientific) and subsequently added to 2 × 10^7^ cells (cell number calculated to maintain library complexities) at a MOI of 0.01 with 2 μg/mL polybrene to initiate the next selection round. This process was conducted in an iterative and parallel fashion for four rounds to select out viral species containing mutations that deleteriously effect replication capacity. Supernatant p24 levels were used to estimate MOI. p24 provides a measurement of viral particle concentration regardless of potential infectivity, often providing an inflated value for MOI calculation. We compared the p24 derived MOI calculation with a tissue culture infectious dose (TCID) limited dilution assay and determined the discrepancy between final values was generally less than one log (p24 > TCID). For all library selection rounds we maintained a low p24 calculated MOI (0.01) to minimize possible trans-complementation between viral variants in the same infected cell. Although this MOI can be considered ≤0.01, further reducing possible trans-complementation, it was not viewed as an experimental obstruction as cell numbers for each selection round were maintained in surplus to assure coverage of the starting library complexities. For each passage, at 24 hours post infection, cells were centrifuged, PBS washed, and re-suspended in fresh RPMI media to remove unadsorbed virus. HIV-1 induced cytopathic effects were visually monitored, and each selection round was typically terminated ~7-10 days post infection. Virion RNA was isolated from cell culture supernatants using QIAamp Viral RNA kit (Qiagen) and reverse transcribed to cDNA using Superscript III Reverse Transcriptase (Invitrogen) using random hexamers. cDNA was then quantified using sybr green qPCR with known concentrations of linearized pNL4-3 plasmid as standards and primers specific to the env gp41 region of the viral genome validated for efficiency previously (m = −3.3, R^2^ = 0.9985), on a DNA engine Opticon 2 real-time cycler (BioRad), using cycle parameters 95°C 3 min, 95°C 20 sec, 56°C 20 sec, 72°C 45 sec, 40 cycles, 72°C for 10 min, and a final hold at 4°C, and data was further used to calculate transcript count.

### Next-generation sequencing of virus mutants

We conceptually designed a two-step PCR strategy to prepare isolated viral RNA (cDNA) after each selection round that is specific for the NGS Illumina HiSeq 2000 platform. Virion cDNA was used as template to amplify amplicons that are ~188 nts using HIV-1 specific primer pairs. The primer space between library fragments 1–7 was constant and therefore not covered in mutagenic selection: primer space 1–2: nucleotides 705–724, 2–3: nucleotides 1995–2022, 3–4: nucleotides 3477–3500, 4–5: 5733–5760, 5–6: 7244–7268, and 6–7: 8878–8898. Each primer pair (69 staggered pairs for genome covered – nucleotides 147–9606, LTR regions 1–146 and 9607–9709 of 5’ and 3’ ends of genome not sequenced) contains a unique nucleotide tag among them consisting of 10 random nucleotides to identify the specific amplicon fragment combined with either two keto bases “K” (T or G) or two amino bases “M” (C or A) to identify the DNA input or R2, respectively. The total number of possible unique nucleotide tag sequences is 4,194,304, ensuring that each individual amplicon in the pool has a unique identifying sequence, and importantly was diverse enough to cover both WT and mutant species. The primer pairs also contain part of the 5’ and 3’ Illumina adapter regions required for sequencing at their termini. For all HIV-1 specific amplicon PCR reactions of step one we used high-fidelity KOD DNA polymerase (EMD Millipore) with the cycle parameters 95°C 2 min, 95°C 20 sec, 56°C 20 sec, 68°C 45 sec, 40 cycles, 68°C for 10 min, and a final hold at 4°C, with the exception of fragment 1 amplicon 5, and fragment 2 amplicon 1, which utilized the annealing temperature of 66°C. Once HIV-1 amplicon fragments were amplified from each round, an aliquot (~5 μL) was electrophoresed on a 3% agarose gel to confirm PCR product amplification. Aliquots (~5 μL) of each HIV-1 amplicon product required for one NGS Illumina HiSeq 2000 sequencing lane were pooled and spun through a PureLink PCR purification column (Invitrogen). We have overcome an inherent NGS error-correction issue by ensuring ten copies of each amplicon is sequenced in order to distinguish mutation versus sequencing error. Based on manufacture information at the time we conducted our experiment the error rate for the HiSeq 2000 NGS platform ranged from 0.1-1%, whereas the typical output per lane of the instrument was ≥150 million filtered reads. For all our calculations, we conservatively estimated the filtered read output per lane at 120 million reads to ensure we obtained sufficient coverage per amplicon. In previous optimization trial experiments using the instrument we directly observed the error rate as low as 0.1%, a rate that still poses a significant challenge to accurately calling true mutations versus instrument error in such a large diverse mutant population. To effectively identify instrument errors, we precisely quantified the number of pooled amplicon molecules from PCR step one, and subsequently decreased the amplicon number to 12 million molecules (typically a dilution of ~12,500X) before it is used as template for the Illumina specific PCR in step two ensuring that a median of 10 copies of each amplicon are present after a sub-saturation (18–20 cycles) PCR.

A cluster containing only three or less reads were filtered removed. In addition, only a mismatch that had an occurrence of >95% within a cluster was called as a true mutation. This criteria provided a high statistical confidence with a p-value ≤ 10^−9^ (binomial exact test) for individual mutation calling. One potential pitfall was that it is possible to have two or more WT copies carrying the same unique nucleotide tag as input for the second step PCR. This would result in an underestimation of WT copy number. Nonetheless, the input copy number for the second PCR was estimated to be ~85,000, which is 50-fold lower than the unique nucleotide tag complexity. The possibility of having any two different molecules carrying the exactly same unique nucleotide tag would be ~0.02% (approximated by Poisson distribution, λ = 85,000/4,194,304). Therefore, the underestimation of WT copy number is very minimal.

Using this approach the sensitivity to detect rare variants was dependent on amplicon coverage, and therefore varied by amplicon and selection round (Additional file 4). For sensitivity, we typically achieved the ability to detect mutants as rare as 0.001% in the viral population after error correction. With the exception of amplicon F1-A5 (nucleotides 568–704), where coverage was consistently low for both input and R2 (sensitivity range of 0.009-0.01%), and amplicon F1-A4 (nucleotides 417–567), where coverage was low in R2 (sensitivity of 0.06%), our sensitivity range to detect rare mutations was 0.0004-0.001% and 0.0008-0.009%, for DNA input and R2, respectively.

Another important aspect to consider in planning our high-scale mutation experiment in achieving a sensitivity after NGS sequencing is to determine true mutation frequency changes above what may be imparted by the cDNA synthesis error rate at 3.4 × 10^−5^. We determined the number of library mutations achieving an input DNA frequency (mutation coverage/amplicon coverage) greater than the reported cDNA synthesis error rate. As can be seen in Additional file 9, frequency of engineered mutations in our library predominately achieves a log scale fold-increase above the cDNA synthesis error rate.

The purified PCR step one amplicon pool was measured via nanodrop, exact DNA molecules calculated, and diluted appropriately to 12 million molecules per lane for error-correction (~12,500X). A single sub-saturation PCR to add on the final regions of the Illumina adapter region was then conducted on the diluted PCR product using high-fidelity KOD DNA polymerase (EMD Millipore) with the cycle parameters 95°C 2 min, 95°C 20 sec, 62°C 20 sec, 68°C 45 sec, 20 cycles, 68°C for 10 min, and a final hold at 4°C. Product from the second PCR was spun through a PureLink PCR purification column (Invitrogen), eluted in dH_2_O, and a 15 μL aliquot at ~8 ng/μL was provided to the DNA Microarray Core Facility at UCLA, where the concentration was confirmed by Qubit, the size and quality confirmed using a Bioanalyzer (Agilent Technologies), and subsequently sequenced on an Illumina HiSeq2000 machine using paired-end 2 × 100 read parameters. Raw sequencing data were deposited to the NCBI Sequence Read Archive (SRA) under accession code BioProject PRJNA259391.

Recently, similar NGS error-correction approaches of ensuring redundancy of unique identifying sequences was independently reported as a means to identify rare cellular mutations and variants within a single gene focused pool [7,36]. The study by Jabara et al. used an 8-mer degenerate nucleotide sequence at the cDNA synthesis step to uniquely identify patient derived HIV-1 PR variants [36]. Unique sequence tagging at the cDNA synthesis step would prove highly beneficial as cDNA synthesis errors could also be correctly identified. However the approach is less amenable to large scale sequencing projects of high gene diversity and is more suitable for targeted gene variant pools. Although these studies share a similar philosophy to overcome the NGS error-correction issue for the detection of rare variants, our study includes a further restraint in precisely limiting the input tagged template copy number and PCR efficiency during the PCR of step two to accurately control the distribution of cluster count in the sequencing output to a median cluster size of 10 amplicons. Limiting redundancy input in order to minimize unnecessary loss of sequencing capacity is also mentioned in a recent NGS error-correction study by Schmitt et al. where the approach was to independently affix a 12-mer unique sequence tag to both strands of a sheared size-selected targeted sequence DNA [37]. After NGS sequencing and error correction, the approximated error frequency was reported at 3.8 × 10^−10^, representing a great sensitivity improvement in rare mutation identification. In this approach the target sequence DNA was sheared and size-selected, an approach more suitable for cellular DNA versus short viral genome DNA fragments, as we observe DNA shearing to over-represent DNA termini. As many NGS error-correction methods are currently being reported in the literature, the goals of the experiment must be evaluated and a suitable error-correction approach selected as each has their applications, limitations, and advantages.

### Sequencing data analysis

Sequencing reads were mapped by Burrows-Wheeler Alignment tool (BWA) [38]. Custom Python scripts were used to match nucleotide tags, conflate error-corrected amplicon sequences, and other downstream analyses. The mutation frequency after selection was determined by dividing the mutation occurrence by the total population count (WT plus variant), whereas the change in frequency was determined by calculating: [R2 frequency/input frequency] of each mutation.

### HIV-1 individual mutant construction

All site directed mutagenesis was conducted with a two-step PCR approach specific to the HIV-1 genomic fragment that contained the targeted substitution. Each substitution and corresponding HIV-1 fragment are as follows: CA A194T:Frag2, PR D25G:Frag3, PR D29G:Frag3, RT E6K:Frag3, RT F61S:Frag3, RT Y501C:Frag4, IN N155Y:Frag4, vif D101N:Frag4, rev E10G:Frag5, gp120 C119G:Frag5, gp120 K205M:Frag5, gp120 D476V:Frag6, gp41 Y136H:Frag6, and 3’LTR C9547T:Frag7. Using 5 ng of pNL4-3 as template, each forward and reverse mutagenic primer was combined with the reverse and forward fragment primers (initially used for error-prone PCR) to generate partial, yet overlapping (at mutagenized codon) PCR fragments of the full sized fragment using high-fidelity KOD DNA polymerase (EMD Millipore) with the cycle parameters 95°C 2 min, 95°C 20 sec, 56°C 20 sec, 68°C 1 min, 30 cycles, 68°C for 10 min, and a final hold at 4°C. Afterwards 5 μL of each purified mutagenic product was combined in a second PCR using the same conditions and cycle parameters with only the forward and reverse fragment primers to generate the full length fragment containing the mutagenized codon. The products were digested with restriction enzymes specific to the fragment: Frag2: BssHII and ApaI, Frag3: ApaI and AgeI, Frag4: AgeI and EcoRI, Frag5: EcoRI and NheI, Frag6: NheI and XhoI, and Frag7: XhoI and NcoI (New England BioLabs), and ligated in correspondingly digested cloning vectors using T4 DNA ligase (Invitrogen) according to manufacturer’s instructions. Mutations were confirmed by sequencing and plasmids were midi-prepped (Invitrogen).

## Additional files

Additional file 1:
**Schematic view of our approach to minimize epistatic effects other mutations have on our mutation of interest RC calculation.** We achieved a very high sequencing coverage per mutation of interest (M) which we hypothesized would average out potential epistatic effects of mutations present on the same mutagenic Kb fragment. Each line represents the same Kb DNA fragment, with mutation of interest, M, represented by open circle, mutations associated with M on each unique DNA molecule are represented by a closed circle. The average RC value of all unique genomes containing mutation M is estimated to be main RC of mutation M. Additional file 2:
**Profile transcript count and amplicon coverage for HIV-1 DNA input and R2 mutant libraries.** (A) HIV-1 proviral transcript count per uL for each single nucleotide point library 1–7 (from bottom to top) for DNA input and R2. Transcript levels for each were high enough to maintain the full complexity of each starting library, and therefore guaranteed we could accurately quantify relative frequencies of each variant. (B) Amplicon coverage of DNA input and R2. Occurrence of each amplicon listed on vertical y-axis, whereas amplicon identity listed on x-axis. Additional file 3:
**Complete cell culture passage scheme for each HIV-1 point mutation library for NGS sample preparation.** Each HIV-1 mutant plasmid library was first reconstituted into viral libraries via 293T transfection and subsequently passaged for two iterative rounds in 20 million CEM T-lymphocytes at a MOI of 0.01. Plasmid libraries (input) and virion cDNA from R2 (output) were used as material for NGS sample preparation. Additional file 4:
**Table of HIV-1 amplicon coverage and mutant sensitivity.**
Additional file 5:
**Profiling reproducibility analysis of overlapping amplicons.** We observed ~90% correlation for mutations existing on overlapping amplicon regions for both the input DNA and R2 selection round. Arbitrarily termed ‘amplicon 1’ and ‘amplicon 2’ in graphs. Additional file 6:
**Table of RC index values for all HIV-1 substitutions.**
Additional file 7:
**Table of qHRG profile results in comparison with HIV-1 integrase viral phenotypes reported in literature.**
Additional file 8:
**Table of oligonucleotides used in this study.**
Additional file 9:
**Frequency of engineered mutations in our libraries predominately achieves a log scale fold-increase above the cDNA synthesis error rate.** Number of library mutations (y-axis) achieving an input DNA frequency (mutation coverage/amplicon coverage) greater than the cDNA synthesis error rate (3.4 × 10^−5^), shown as dashed red line (set as base line log_10_ fold change = 0), in log scale fold-increase (x-axis).
