# Supplementary figures and images for: High-throughput profiling of point mutations across the HIV-1 genome
Source: Retrovirology. 2014 Dec 19;11:124. doi: 10.1186/s12977-014-0124-6 (PMC4300175; doi:10.1186/s12977-014-0124-6)

## Slide 1
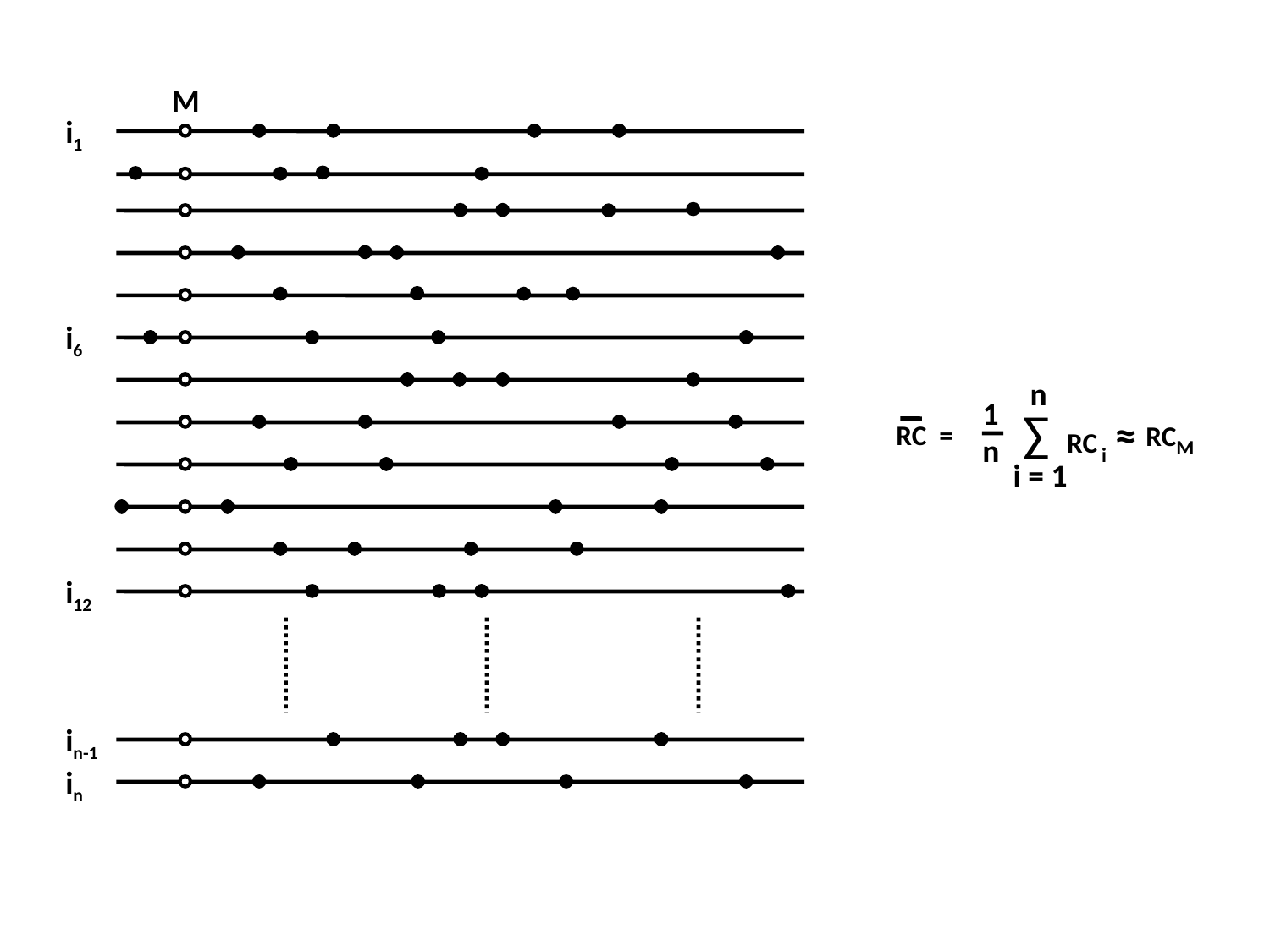

M
i1
i6
n
1
∑
≈
RCM
RC =
RC i
n
i = 1
i12
in-1
in

Supplement: Additional file 1: — Schematic view of our approach to minimize epistatic effects other mutations have on our mutation of interest RC calculation. We achieved a very high sequencing coverage per mutation of interest (M) which we hypothesized would average out potential epistatic effects of mutations present on the same mutagenic Kb fragment. Each line represents the same Kb DNA fragment, with mutation of interest, M, represented by open circle, mutations associated with M on each unique DNA molecule are represented by a closed circle. The average RC value of all unique genomes containing mutation M is estimated to be main RC of mutation M. [file 12977_2014_124_MOESM1_ESM.ppt]

## Slide 1
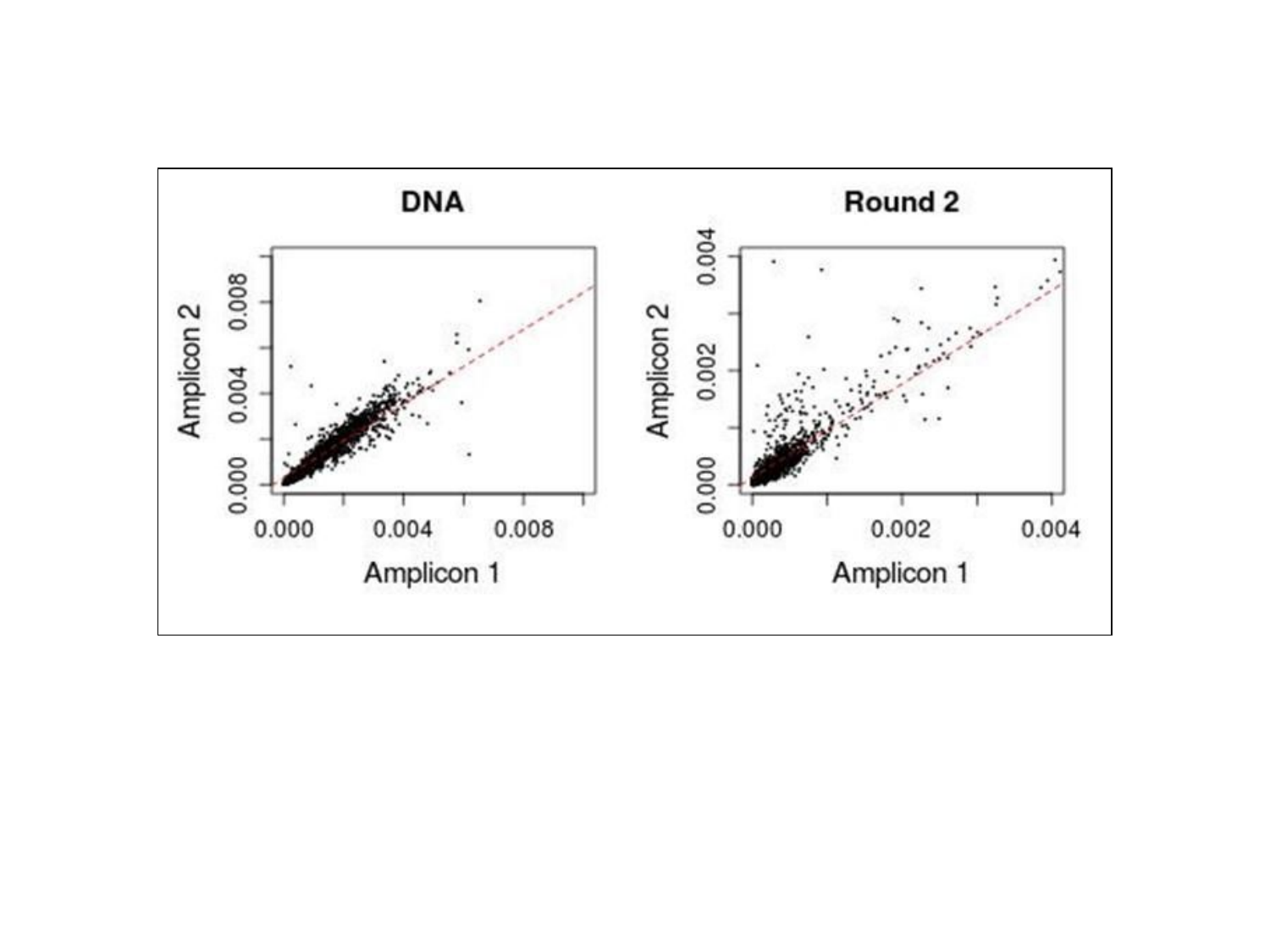

Supplement: Additional file 5: — Profiling reproducibility analysis of overlapping amplicons. We observed ~90% correlation for mutations existing on overlapping amplicon regions for both the input DNA and R2 selection round. Arbitrarily termed ‘amplicon 1’ and ‘amplicon 2’ in graphs. [file 12977_2014_124_MOESM5_ESM.ppt]

## Slide 1
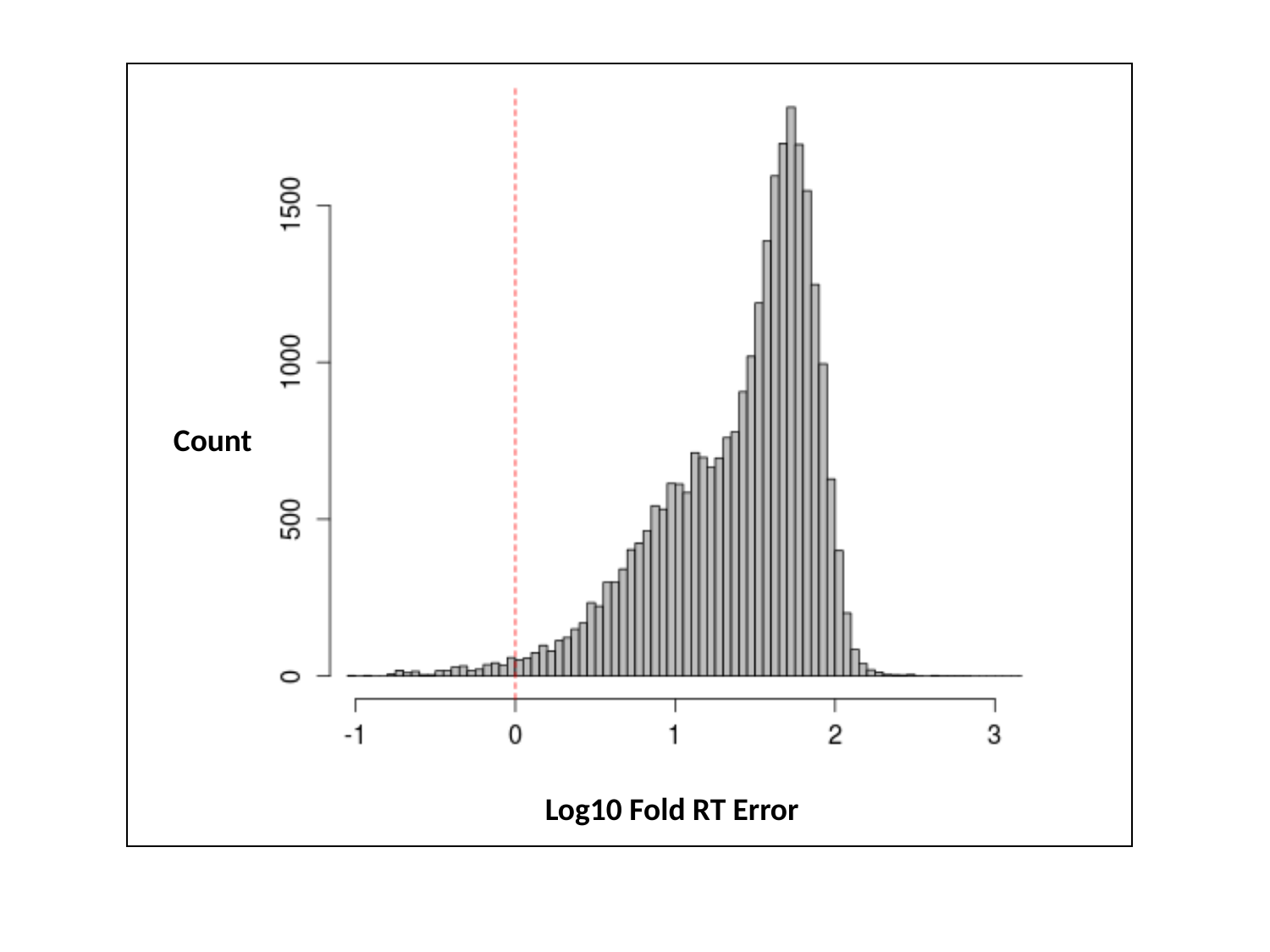

Count
Log10 Fold RT Error

Supplement: Additional file 9: — Frequency of engineered mutations in our libraries predominately achieves a log scale fold-increase above the cDNA synthesis error rate. Number of library mutations (y-axis) achieving an input DNA frequency (mutation coverage/amplicon coverage) greater than the cDNA synthesis error rate (3.4 × 10^−5^), shown as dashed red line (set as base line log10 fold change = 0), in log scale fold-increase (x-axis). [file 12977_2014_124_MOESM9_ESM.ppt]
