# Supplementary material for: High-throughput profiling of point mutations across the HIV-1 genome
Source: Retrovirology. 2014 Dec 19;11:124. doi: 10.1186/s12977-014-0124-6 (PMC4300175; doi:10.1186/s12977-014-0124-6)
Supplement: Additional file 2: — Profile transcript count and amplicon coverage for HIV-1 DNA input and R2 mutant libraries. (A) HIV-1 proviral transcript count per uL for each single nucleotide point library 1–7 (from bottom to top) for DNA input and R2. Transcript levels for each were high enough to maintain the full complexity of each starting library, and therefore guaranteed we could accurately quantify relative frequencies of each variant. (B) Amplicon coverage of DNA input and R2. Occurrence of each amplicon listed on vertical y-axis, whereas amplicon identity listed on x-axis. [file 12977_2014_124_MOESM2_ESM.ppt]

## Slide 1
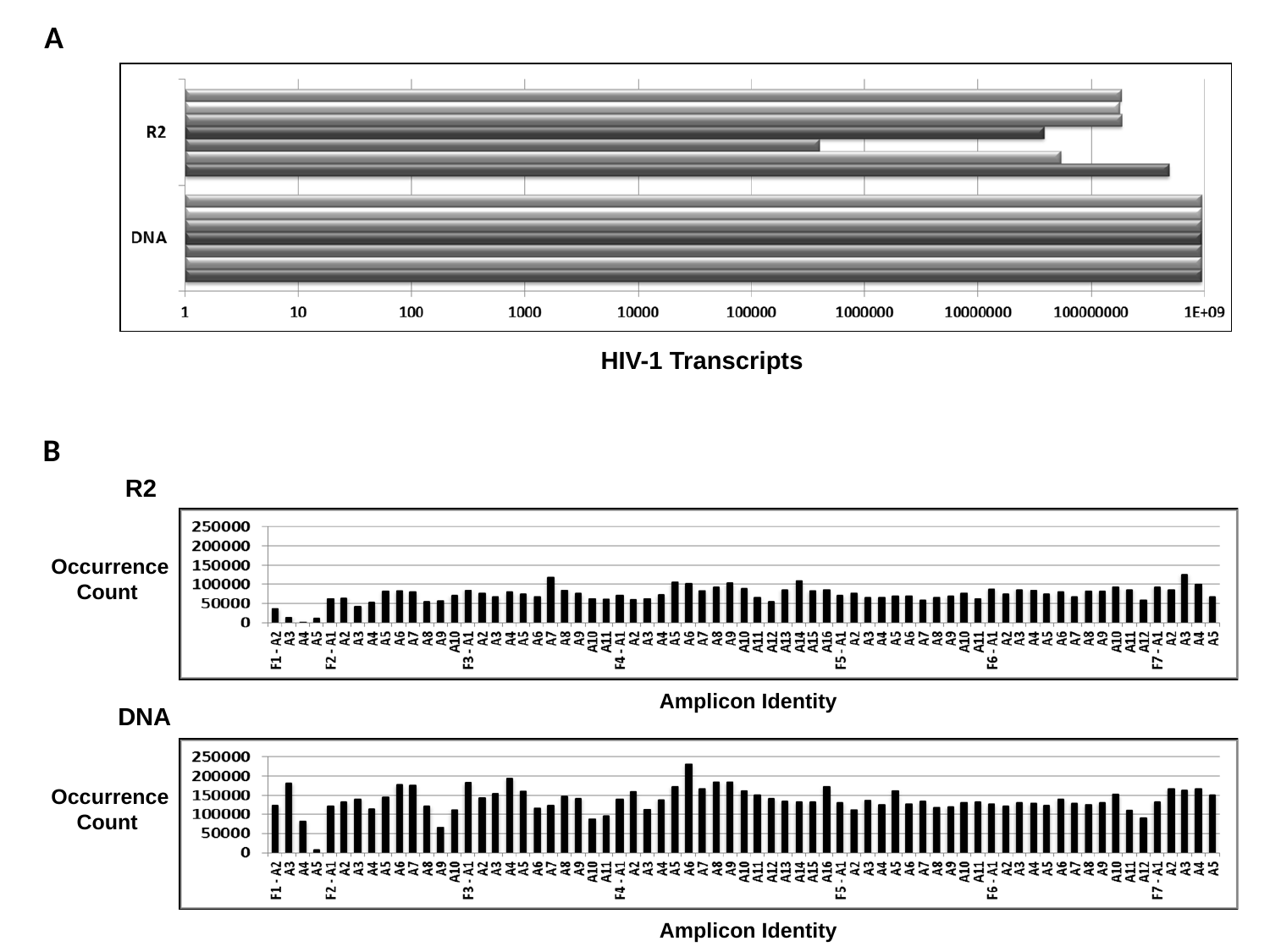

A
HIV-1 Transcripts
B
R2
Occurrence Count
Amplicon Identity
DNA
Occurrence Count
Amplicon Identity
