# Supplementary material for: High-throughput profiling of point mutations across the HIV-1 genome
Source: Retrovirology. 2014 Dec 19;11:124. doi: 10.1186/s12977-014-0124-6 (PMC4300175; doi:10.1186/s12977-014-0124-6)
Supplement: Additional file 3: — Complete cell culture passage scheme for each HIV-1 point mutation library for NGS sample preparation. Each HIV-1 mutant plasmid library was first reconstituted into viral libraries via 293T transfection and subsequently passaged for two iterative rounds in 20 million CEM T-lymphocytes at a MOI of 0.01. Plasmid libraries (input) and virion cDNA from R2 (output) were used as material for NGS sample preparation. [file 12977_2014_124_MOESM3_ESM.ppt]

## Slide 1
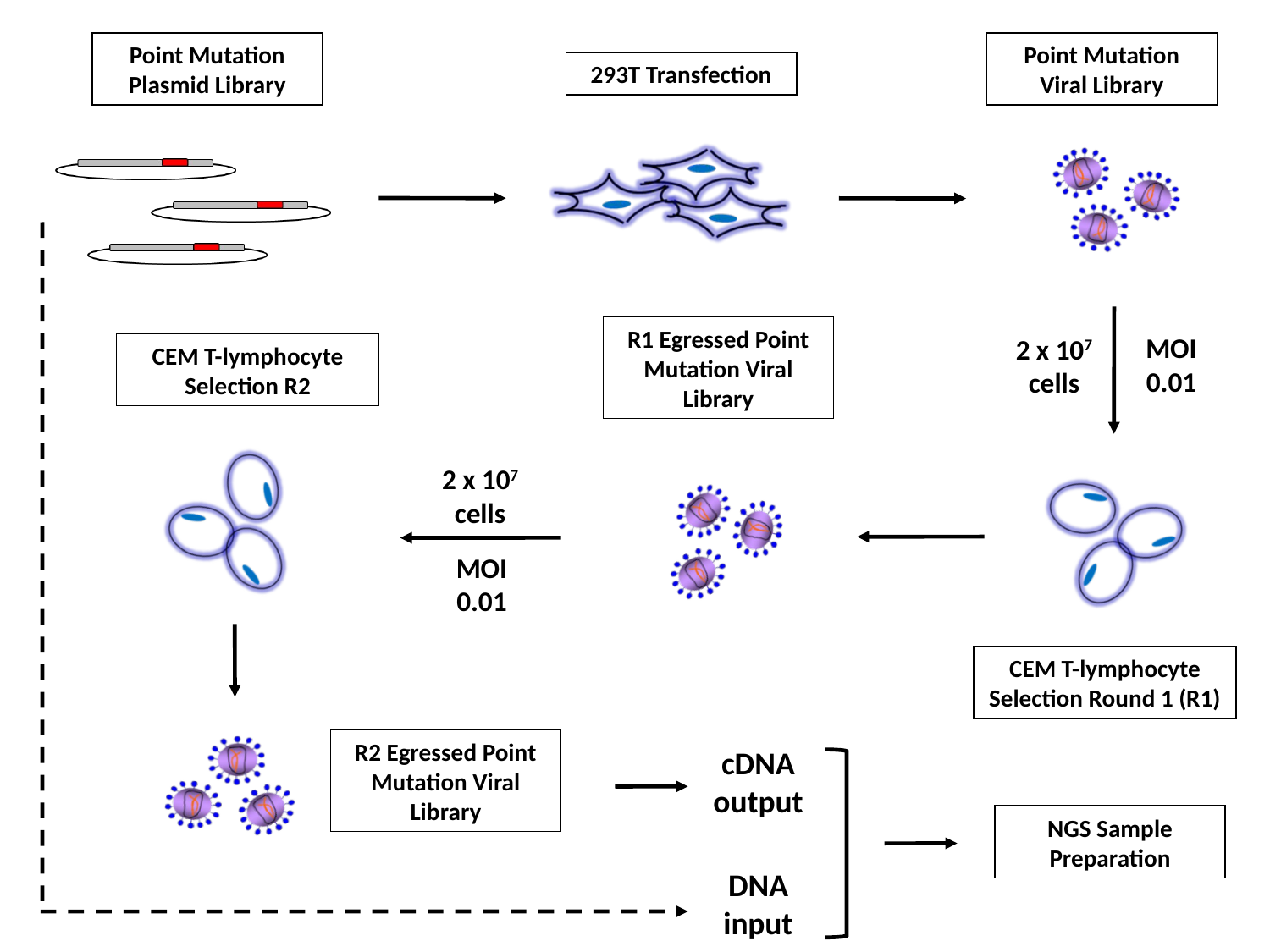

Point Mutation Plasmid Library
Point Mutation Viral Library
293T Transfection
R1 Egressed Point Mutation Viral Library
MOI 0.01
2 x 10^7^ cells
CEM T-lymphocyte Selection R2
2 x 10^7^ cells
MOI 0.01
CEM T-lymphocyte Selection Round 1 (R1)
R2 Egressed Point Mutation Viral Library
cDNA output
NGS Sample Preparation
DNA input
