# Supplementary material for: High-throughput profiling of point mutations across the HIV-1 genome
Source: Retrovirology. 2014 Dec 19;11:124. doi: 10.1186/s12977-014-0124-6 (PMC4300175; doi:10.1186/s12977-014-0124-6)
Supplement: Additional file 4: — Table of HIV-1 amplicon coverage and mutant sensitivity. [file 12977_2014_124_MOESM4_ESM.docx]

(*Retrovirology*)

**High-throughput Profiling of Point Mutations across the HIV-1 Genome**

Laith Q. Al-Mawsawi^1,2^, Nicholas C. Wu^1,3^, C. Anders Olson^1^, Vivian Cai Shi^1^, Hangfei Qi^1^, Xiaojuan Zheng^1^, Ting-Ting Wu^1^, and Ren Sun^1,2,3*^

| **Additional file 4. Table of HIV-1 amplicon coverage and mutant sensitivity** | | | | |
| --- | --- | --- | --- | --- |
|  | **Plasmid DNA** | | **Selection Round 2** | |
| **Amplicon** | **C** | **S** | **C** | **S** |
| **F1** |  |  |  |  |
| **A2** | 123977 | 8.07E-06 | 36353 | 2.75E-05 |
| **A2/A3** | 305703 | 3.27E-06 | 51170 | 1.95E-05 |
| **A3** | 181726 | 5.5E-06 | 14817 | 6.75E-05 |
| **A3/A4** | 263283 | 3.8E-06 | 16507 | 6.06E-05 |
| **A4** | 81557 | 1.23E-05 | 1690 | 0.000592 |
| **A5** | 8067 | 0.000124 | 10773 | 9.28E-05 |
| **F2** |  |  |  |  |
| **A1** | 121353 | 8.24E-06 | 62789 | 1.59E-05 |
| **A1/A2** | 253593 | 3.94E-06 | 128050 | 7.81E-06 |
| **A2** | 132240 | 7.56E-06 | 65261 | 1.53E-05 |
| **A3** | 140843 | 7.1E-06 | 43816 | 2.28E-05 |
| **A3/A4** | 255977 | 3.91E-06 | 96241 | 1.04E-05 |
| **A4** | 115134 | 8.69E-06 | 52425 | 1.91E-05 |
| **A4/A5** | 261050 | 3.83E-06 | 134597 | 7.43E-06 |
| **A5** | 145916 | 6.85E-06 | 82172 | 1.22E-05 |
| **A5/A6** | 323598 | 3.09E-06 | 164833 | 6.07E-06 |
| **A6** | 177682 | 5.63E-06 | 82661 | 1.21E-05 |
| **A6/A7** | 353985 | 2.82E-06 | 162998 | 6.14E-06 |
| **A7** | 176303 | 5.67E-06 | 80337 | 1.24E-05 |
| **A7/A8** | 299052 | 3.34E-06 | 134587 | 7.43E-06 |
| **A8** | 122749 | 8.15E-06 | 54250 | 1.84E-05 |
| **A8/A9** | 189080 | 5.29E-06 | 110986 | 9.01E-06 |
| **A9** | 66331 | 1.51E-05 | 56736 | 1.76E-05 |
| **A9/A10** | 179208 | 5.58E-06 | 128538 | 7.78E-06 |
| **A10** | 112877 | 8.86E-06 | 71802 | 1.39E-05 |
| **F3** |  |  |  |  |
| **A1** | 181887 | 5.5E-06 | 85821 | 1.17E-05 |
| **A2** | 144723 | 6.91E-06 | 76648 | 1.3E-05 |
| **A2/A3** | 298982 | 3.34E-06 | 145412 | 6.88E-06 |
| **A3** | 154259 | 6.48E-06 | 68764 | 1.45E-05 |
| **A3/A4** | 349499 | 2.86E-06 | 148460 | 6.74E-06 |
| **A4** | 195240 | 5.12E-06 | 79696 | 1.25E-05 |
| **A4/A5** | 356673 | 2.8E-06 | 155433 | 6.43E-06 |
| **A5** | 161433 | 6.19E-06 | 75737 | 1.32E-05 |
| **A5/A6** | 278681 | 3.59E-06 | 144585 | 6.92E-06 |
| **A6** | 117248 | 8.53E-06 | 68848 | 1.45E-05 |
| **A7** | 123658 | 8.09E-06 | 118815 | 8.42E-06 |
| **A8** | 148220 | 6.75E-06 | 85939 | 1.16E-05 |
| **A9** | 142805 | 7E-06 | 75994 | 1.32E-05 |
| **A10** | 88452 | 1.13E-05 | 62492 | 1.6E-05 |
| **A10/A11** | 186173 | 5.37E-06 | 124085 | 8.06E-06 |
| **A11** | 97721 | 1.02E-05 | 61593 | 1.62E-05 |
| **F4** |  |  |  |  |
| **A1** | 140219 | 7.13E-06 | 72378 | 1.38E-05 |
| **A2** | 158996 | 6.29E-06 | 59904 | 1.67E-05 |
| **A3** | 113979 | 8.77E-06 | 62453 | 1.6E-05 |
| **A4** | 139426 | 7.17E-06 | 73369 | 1.36E-05 |
| **A5** | 173301 | 5.77E-06 | 105736 | 9.46E-06 |
| **A6** | 230957 | 4.33E-06 | 102442 | 9.76E-06 |
| **A6/A7** | 397907 | 2.51E-06 | 186060 | 5.37E-06 |
| **A7** | 166950 | 5.99E-06 | 83618 | 1.2E-05 |
| **A8** | 185128 | 5.4E-06 | 93600 | 1.07E-05 |
| **A8/A9** | 369060 | 2.71E-06 | 196607 | 5.09E-06 |
| **A9** | 183932 | 5.44E-06 | 103007 | 9.71E-06 |
| **A9/A10** | 346261 | 2.89E-06 | 193111 | 5.18E-06 |
| **A10** | 162329 | 6.16E-06 | 90104 | 1.11E-05 |
| **A11** | 150329 | 6.65E-06 | 66987 | 1.49E-05 |
| **A12** | 141678 | 7.06E-06 | 55406 | 1.8E-05 |
| **A13** | 134611 | 7.43E-06 | 86653 | 1.15E-05 |
| **A14** | 132242 | 7.56E-06 | 109570 | 9.13E-06 |
| **A14/A15** | 263938 | 3.79E-06 | 192801 | 5.19E-06 |
| **A15** | 131696 | 7.59E-06 | 83231 | 1.2E-05 |
| **A15/A16** | 303984 | 3.29E-06 | 169296 | 5.91E-06 |
| **A16** | 172288 | 5.8E-06 | 86065 | 1.16E-05 |
| **F5** |  |  |  |  |
| **A1** | 130505 | 7.66E-06 | 72117 | 1.39E-05 |
| **A1/A2** | 243165 | 4.11E-06 | 148999 | 6.71E-06 |
| **A2** | 112660 | 8.88E-06 | 76882 | 1.3E-05 |
| **A2/A3** | 249653 | 4.01E-06 | 142871 | 7E-06 |
| **A3** | 136993 | 7.3E-06 | 65989 | 1.52E-05 |
| **A3/A4** | 261874 | 3.82E-06 | 132191 | 7.56E-06 |
| **A4** | 124881 | 8.01E-06 | 66202 | 1.51E-05 |
| **A5** | 162285 | 6.16E-06 | 69301 | 1.44E-05 |
| **A5/A6** | 288919 | 3.46E-06 | 140003 | 7.14E-06 |
| **A6** | 126634 | 7.9E-06 | 70702 | 1.41E-05 |
| **A6/A7** | 260304 | 3.84E-06 | 129386 | 7.73E-06 |
| **A7** | 133670 | 7.48E-06 | 58684 | 1.7E-05 |
| **A7/A8** | 251651 | 3.97E-06 | 124957 | 8E-06 |
| **A8** | 117981 | 8.48E-06 | 66273 | 1.51E-05 |
| **A9** | 120607 | 8.29E-06 | 69430 | 1.44E-05 |
| **A10** | 130688 | 7.65E-06 | 76630 | 1.3E-05 |
| **A10/A11** | 262111 | 3.82E-06 | 139932 | 7.15E-06 |
| **A11** | 131423 | 7.61E-06 | 63302 | 1.58E-05 |
| **F6** |  |  |  |  |
| **A1** | 126529 | 7.9E-06 | 88141 | 1.13E-05 |
| **A1/A2** | 249498 | 4.01E-06 | 162528 | 6.15E-06 |
| **A2** | 122969 | 8.13E-06 | 74387 | 1.34E-05 |
| **A3** | 131082 | 7.63E-06 | 87086 | 1.15E-05 |
| **A3/A4** | 259309 | 3.86E-06 | 172445 | 5.8E-06 |
| **A4** | 128227 | 7.8E-06 | 85359 | 1.17E-05 |
| **A5** | 123442 | 8.1E-06 | 75704 | 1.32E-05 |
| **A6** | 139920 | 7.15E-06 | 80367 | 1.24E-05 |
| **A7** | 129544 | 7.72E-06 | 68683 | 1.46E-05 |
| **A7/A8** | 254875 | 3.92E-06 | 150809 | 6.63E-06 |
| **A8** | 125331 | 7.98E-06 | 82126 | 1.22E-05 |
| **A9** | 130336 | 7.67E-06 | 80957 | 1.24E-05 |
| **A10** | 152296 | 6.57E-06 | 92835 | 1.08E-05 |
| **A11** | 110321 | 9.06E-06 | 87303 | 1.15E-05 |
| **A11/A12** | 202162 | 4.95E-06 | 145994 | 6.85E-06 |
| **A12** | 91841 | 1.09E-05 | 58691 | 1.7E-05 |
| **F7** |  |  |  |  |
| **A1** | 132535 | 7.55E-06 | 93945 | 1.06E-05 |
| **A2** | 167515 | 5.97E-06 | 87109 | 1.15E-05 |
| **A3** | 164187 | 6.09E-06 | 125981 | 7.94E-06 |
| **A4** | 168315 | 5.94E-06 | 99669 | 1E-05 |
| **A4/A5** | 319796 | 3.13E-06 | 167947 | 5.95E-06 |
| **A5** | 151481 | 6.6E-06 | 68278 | 1.46E-05 |
