# Supplementary material for: High-throughput profiling of point mutations across the HIV-1 genome
Source: Retrovirology. 2014 Dec 19;11:124. doi: 10.1186/s12977-014-0124-6 (PMC4300175; doi:10.1186/s12977-014-0124-6)
Supplement: Additional file 7: — Table of qHRG profile results in comparison with HIV-1 integrase viral phenotypes reported in literature. [file 12977_2014_124_MOESM7_ESM.docx]

(*Retrovirology*)

**High-throughput Profiling of Point Mutations across the HIV-1 Genome**

Laith Q. Al-Mawsawi^1,2^, Nicholas C. Wu^1,3^, C. Anders Olson^1^, Vivian Cai Shi^1^, Hangfei Qi^1^, Xiaojuan Zheng^1^, Ting-Ting Wu^1^, and Ren Sun^1,2,3*^

| **Additional file 7.** **Table of qHRG profile results in comparison with HIV-1 integrase viral phenotypes reported in literature** | | | | |
| --- | --- | --- | --- | --- |
| **Integrase substitution** | **Reported phenotype** | **Reference** | **RC index** | **Agreement** |
| D64E | lethal | [1,2] | 0.039 | + |
| T66A | non-lethal | [1,3] | 0.139 | + |
| S81I | lethal | [1] | 0.048 | + |
| S81R | lethal | [4] | 0.019 | + |
| K111E | non-lethal | [5] | 0.043 | - |
| T115A | non-lethal | [3,6] | 0.103 | + |
| N117K | delayed RC^a^ | [6,7] | 0.05 | + |
| K136E | non-lethal | [7,8] | 0.046 | - |
| E138K | non-lethal | [7] | 0.604 | + |
| Y143N | delayed RC^a^ | [6] | 0.031 | + |
| N144K | delayed RC^a^ | [7] | 0.035 | + |
| E152D | lethal | [7] | 0.037 | + |
| E152G | lethal | [4] | 0.039 | + |
| K156E | lethal | [9] | 0.057 | + |
| V165A | lethal | [10] | 0.126 | - |
| N184D | lethal | [7] | 0.052 | + |
| F185L | lethal | [8] | 0.053 | + |
| K236E | lethal | [11] | 0.091 | + |
| V260E | lethal | [11] | 0.057 | + |
| K266E | lethal | [11] | 0.16 | - |
| 1. Taddeo B, Haseltine WA, Farnet CM (1994) Integrase mutants of human immunodeficiency virus type 1 with a specific defect in integration. J Virol 68: 8401-8405.  2. Masuda T, Planelles V, Krogstad P, Chen IS (1995) Genetic analysis of human immunodeficiency virus type 1 integrase and the U3 att site: unusual phenotype of mutants in the zinc finger-like domain. J Virol 69: 6687-6696.  3. Cannon PM, Wilson W, Byles E, Kingsman SM, Kingsman AJ (1994) Human immunodeficiency virus type 1 integrase: effect on viral replication of mutations at highly conserved residues. J Virol 68: 4768-4775.  4. Leavitt AD, Robles G, Alesandro N, Varmus HE (1996) Human immunodeficiency virus type 1 integrase mutants retain in vitro integrase activity yet fail to integrate viral DNA efficiently during infection. J Virol 70: 721-728.  5. Mathew S, Nguyen M, Wu X, Pal A, Shah VB, et al. (2013) INI1/hSNF5-interaction defective HIV-1 IN mutants exhibit impaired particle morphology, reverse transcription and integration in vivo. Retrovirology 10: 66.  6. Shin CG, Taddeo B, Haseltine WA, Farnet CM (1994) Genetic analysis of the human immunodeficiency virus type 1 integrase protein. J Virol 68: 1633-1642.  7. Lu R, Limon A, Ghory HZ, Engelman A (2005) Genetic analysis of DNA-binding mutants in the catalytic core domain of human immunodeficiency virus type 1 integrase. J Virol 79: 2493-2505.  8. Engelman A, Liu Y, Chen H, Farzan M, Dyda F (1997) Structure-based mutagenesis of the catalytic domain of human immunodeficiency virus type 1 integrase. J Virol 71: 3507-3514.  9. Jenkins TM, Esposito D, Engelman A, Craigie R (1997) Critical contacts between HIV-1 integrase and viral DNA identified by structure-based analysis and photo-crosslinking. EMBO J 16: 6849-6859.  10. Limon A, Devroe E, Lu R, Ghory HZ, Silver PA, et al. (2002) Nuclear localization of human immunodeficiency virus type 1 preintegration complexes (PICs): V165A and R166A are pleiotropic integrase mutants primarily defective for integration, not PIC nuclear import. J Virol 76: 10598-10607.  11. Lu R, Ghory HZ, Engelman A (2005) Genetic analyses of conserved residues in the carboxyl-terminal domain of human immunodeficiency virus type 1 integrase. J Virol 79: 10356-10368.  ^a^Single mutant HIV-1 displaying a severely delayed onset in replication would be outcompeted in the qHRG profile by variants with a higher RC within the mutant population and display a lethal phenotype. | | | | |
