# Supplementary material for: High-throughput profiling of point mutations across the HIV-1 genome
Source: Retrovirology. 2014 Dec 19;11:124. doi: 10.1186/s12977-014-0124-6 (PMC4300175; doi:10.1186/s12977-014-0124-6)
Supplement: Additional file 8: — Table of oligonucleotides used in this study. [file 12977_2014_124_MOESM8_ESM.docx]

(*Retrovirology*)

**High-throughput Profiling of Point Mutations across the HIV-1 Genome**

Laith Q. Al-Mawsawi^1,2^, Nicholas C. Wu^1,3^, C. Anders Olson^1^, Vivian Cai Shi^1^, Hangfei Qi^1^, Xiaojuan Zheng^1^, Ting-Ting Wu^1^, and Ren Sun^1,2,3*^

| **Additional file 8. Table of oligonucleotides used in this study** | |
| --- | --- |
| **Error prone fragment PCR** | |
| **Frag1-F** | CCACCTGACGTCTAAGAAACC |
| **Frag1-R** | CTTGCCGTGCGCGCTTCAGC |
| **Frag2-F** | GCTGAAGCGCGCACGGCAAG |
| **Frag2-R** | CTTTTTCCTAGGGGCCCTGCAATTTTTG |
| **Frag3-F** | CAAAAATTGCAGGGCCCCTAGGAAAAAG |
| **Frag3-R** | CACTCCATGTACCGGTTCTTTTAG |
| **Frag4-F** | CTAAAAGAACCGGTACATGGAGTG |
| **Frag4-R** | GCAGTTGTTGCAGAATTCTTATTATGGC |
| **Frag5-F** | GCCATAATAAGAATTCTGCAACAACTGC |
| **Frag5-R** | GTTCTCTTAATTTGCTAGCTATCTG |
| **Frag6-F** | CAGATAGCTAGCAAATTAAGAGAAC |
| **Frag6-R** | CTAGGTCTCGAGATACTGCTC |
| **Frag7-F** | GAGCAGTATCTCGAGACCTAG |
| **Frag7-R** | CCTGCACTCCATGGATCAGC |
| **Cloning Vector Inserts** | |
| **Frag1 VF-F** | Phos - CATCACGCGTATAAG |
| **Frag1 VF-R** | Phos - CGCGCTTATACGCGTGATGACGT |
| **Frag2 VF-F** | Phos - CGCGCATCACGCGTATAAGGGCC |
| **Frag2 VF-R** | Phos - CTTATACGCGTGATG |
| **Frag3 VF-F** | Phos - CTATCACGCGTATAA |
| **Frag3 VF-R** | Phos - CCGGTTATACGCGTGATAGGGCC |
| **Frag4 VF-F** | Phos - CCGGTATCACGCGTATAG |
| **Frag4 VF-R** | Phos - AATTCTATACGCGTGATG |
| **Frag5 VF-F** | Phos - AATTCATCACGCGTATAG |
| **Frag5 VF-R** | Phos - CTAGCTATACGCGTGATG |
| **Frag6 VF-F** | Phos - CTAGCATCACGCGTATAC |
| **Frag6 VF-R** | Phos - TCGAGTATACGCGTGATG |
| **Frag7 VF-F** | Phos - TCGAGATCACGCGTATAC |
| **Frag7 VF-R** | Phos - CATGGTATACGCGTGATC |
| **qPCR transcript quantification - gp41** | |
| **gp41 -F** | ACAGCTCCAGGCAAGAA |
| **gp41 -R** | AGCATTCCAAGGCACAG |
| **NGS preparation PCR 1: HIV-1 specific** | |
| **F1_A2_FK** | CACGACGCTCTTCCGATCTKKNNNNTTGGATGGTGCTTCAAGTTA |
| **F1_A2_FM** | CACGACGCTCTTCCGATCTMMNNNNTTGGATGGTGCTTCAAGTTA |
| **F1_A2_R** | CTGAACCGCTCTTCCGATCTNNNNNNCTC CGG ATG CAG CTC T |
| **F1_A3_FK** | CACGACGCTCTTCCGATCTKKNNNNAGTGTGGAAGTTTGACAGC |
| **F1_A3_FM** | CACGACGCTCTTCCGATCTMMNNNNAGTGTGGAAGTTTGACAGC |
| **F1_A3_R** | CTGAACCGCTCTTCCGATCTNNNNNNGCAGCTGCTTATATGTAGCATC |
| **F1_A4_FK** | CACGACGCTCTTCCGATCTKKNNNNAGTGGCGAGCCCTC |
| **F1_A4_FM** | CACGACGCTCTTCCGATCTMMNNNNAGTGGCGAGCCCTC |
| **F1_A4_R** | CTGAACCGCTCTTCCGATCTNNNNNNTAGTTACCAGAGTCACACAACAG |
| **F1_A5_FK** | CACGACGCTCTTCCGATCTKKNNNNAGTAGTGTGTGCCCGT |
| **F1_A5_FM** | CACGACGCTCTTCCGATCTMMNNNNAGTAGTGTGTGCCCGT |
| **F1_A5_R** | CTGAACCGCTCTTCCGATCTNNNNNNCTTGCCGTGCGCGCTTCAGC |
| **F2_A1_FK** | CACGACGCTCTTCCGATCTKKNNNNGCTGAAGCGCGCACGGCAAG |
| **F2_A1_FM** | CACGACGCTCTTCCGATCTMMNNNNGCTGAAGCGCGCACGGCAAG |
| **F2_A1_R** | CTGAACCGCTCTTCCGATCTNNNNNNTTGTTTCTTTCCCCCTGGCC |
| **F2_A2_FK** | CACGACGCTCTTCCGATCTKKNNNNGCGGGGGAGAATTAGATAA |
| **F2_A2_FM** | CACGACGCTCTTCCGATCTMMNNNNGCGGGGGAGAATTAGATAA |
| **F2_A2_R** | CTGAACCGCTCTTCCGATCTNNNNNNTCCTGTCTGAAGGGATGGTTG |
| **F2_A3_FK** | CACGACGCTCTTCCGATCTKKNNNNGACAAATACTGGGACAGCTA |
| **F2_A3_FM** | CACGACGCTCTTCCGATCTMMNNNNGACAAATACTGGGACAGCTA |
| **F2_A3_R** | CTGAACCGCTCTTCCGATCTNNNNNNAGCTGCTGCTTGCTGTGCC |
| **F2_A4_FK** | CACGACGCTCTTCCGATCTKKNNNNGATAGAGGAAGAGCAAAACAAAA |
| **F2_A4_FM** | CACGACGCTCTTCCGATCTMMNNNNGATAGAGGAAGAGCAAAACAAAA |
| **F2_A4_R** | CTGAACCGCTCTTCCGATCTNNNNNNGGCTGAAAGCCTTCTCTTC |
| **F2_A5_FK** | CACGACGCTCTTCCGATCTKKNNNNCACCTAGAACTTTAAATGCATGGG |
| **F2_A5_FM** | CACGACGCTCTTCCGATCTMMNNNNCACCTAGAACTTTAAATGCATGGG |
| **F2_A5_R** | CTGAACCGCTCTTCCGATCTNNNNNNTGCAGCTTCCTCATTGATGG |
| **F2_A6_FK** | CACGACGCTCTTCCGATCTKKNNNNCATCAAGCAGCCATGCAA |
| **F2_A6_FM** | CACGACGCTCTTCCGATCTMMNNNNCATCAAGCAGCCATGCAA |
| **F2_A6_R** | CTGAACCGCTCTTCCGATCTNNNNNNATAGGTGGATTATGTGTCATCC |
| **F2_A7_FK** | CACGACGCTCTTCCGATCTKKNNNNGGAACTACTAGTACCCTTCAGGA |
| **F2_A7_FM** | CACGACGCTCTTCCGATCTMMNNNNGGAACTACTAGTACCCTTCAGGA |
| **F2_A7_R** | CTGAACCGCTCTTCCGATCTNNNNNNAATCGGTCTACATAGTCTCTAAAGGG |
| **F2_A8_FK** | CACGACGCTCTTCCGATCTKKNNNNGCCCTACCAGCATTCTGG |
| **F2_A8_FM** | CACGACGCTCTTCCGATCTMMNNNNGCCCTACCAGCATTCTGG |
| **F2_A8_R** | CTGAACCGCTCTTCCGATCTNNNNNNTCCTGGTCCCAATGCTTTT |
| **F2_A9_FK** | CACGACGCTCTTCCGATCTKKNNNNTGCGAACCCAGATTGTAAGA |
| **F2_A9_FM** | CACGACGCTCTTCCGATCTMMNNNNTGCGAACCCAGATTGTAAGA |
| **F2_A9_R** | CTGAACCGCTCTTCCGATCTNNNNNNGCCTTTCTGTATCATTATGGTAGC |
| **F2_A10_FK** | CACGACGCTCTTCCGATCTKKNNNNCATGTCAGGGAGTGGGG |
| **F2_A10_FM** | CACGACGCTCTTCCGATCTMMNNNNCATGTCAGGGAGTGGGG |
| **F2_A10_R** | CTGAACCGCTCTTCCGATCTNNNNNNCCTAGGGGCCCTGCAATTTTTG |
| **F3_A1_FK** | CACGACGCTCTTCCGATCTKKNNNNTTGCAGGGCCCCTAGGAAAAAG |
| **F3_A1_FM** | CACGACGCTCTTCCGATCTMMNNNNTTGCAGGGCCCCTAGGAAAAAG |
| **F3_A1_R** | CTGAACCGCTCTTCCGATCTNNNNNNCCCCAAACCTGAAGCTCTCT |
| **F3_A2_FK** | CACGACGCTCTTCCGATCTKKNNNNCCAACAGCCCCACCAGA |
| **F3_A2_FM** | CACGACGCTCTTCCGATCTMMNNNNCCAACAGCCCCACCAGA |
| **F3_A2_R** | CTGAACCGCTCTTCCGATCTNNNNNNATCTGCTCCTGTATCTAATAGAGC |
| **F3_A3_FK** | CACGACGCTCTTCCGATCTKKNNNNCGACCCCTCGTCACAATA |
| **F3_A3_FM** | CACGACGCTCTTCCGATCTMMNNNNCGACCCCTCGTCACAATA |
| **F3_A3_R** | CTGAACCGCTCTTCCGATCTNNNNNNTTATGTCCGCAGATTTCTATGAG |
| **F3_A4_FK** | CACGACGCTCTTCCGATCTKKNNNNTCAAAGTAGGACAGTATGATCAGA |
| **F3_A4_FM** | CACGACGCTCTTCCGATCTMMNNNNTCAAAGTAGGACAGTATGATCAGA |
| **F3_A4_R** | CTGAACCGCTCTTCCGATCTNNNNNNATCCATTCCTGGCTTTAATTTTAC |
| **F3_A5_FK** | CACGACGCTCTTCCGATCTKKNNNNCCCATTAGTCCTATTGAGACTGTAC |
| **F3_A5_FM** | CACGACGCTCTTCCGATCTMMNNNNCCCATTAGTCCTATTGAGACTGTAC |
| **F3_A5_R** | CTGAACCGCTCTTCCGATCTNNNNNNATGGCAAATACTGGAGTATTGTATG |
| **F3_A6_FK** | CACGACGCTCTTCCGATCTKKNNNNTTGTACAGAAATGGAAAAGGAAGG |
| **F3_A6_FM** | CACGACGCTCTTCCGATCTMMNNNNTTGTACAGAAATGGAAAAGGAAGG |
| **F3_A6_R** | CTGAACCGCTCTTCCGATCTNNNNNNACCCTGCAGGATGTGGTAT |
| **F3_A7_FK** | CACGACGCTCTTCCGATCTKKNNNNGATTTCTGGGAAGTTCAATTAGGA |
| **F3_A7_FM** | CACGACGCTCTTCCGATCTMMNNNNGATTTCTGGGAAGTTCAATTAGGA |
| **F3_A7_R** | CTGAACCGCTCTTCCGATCTNNNNNNACATTGTACTGATATCTAATCCCTGG |
| **F3_A8_FK** | CACGACGCTCTTCCGATCTKKNNNNCCATACCTAGTATAAACAATGAGACA |
| **F3_A8_FM** | CACGACGCTCTTCCGATCTMMNNNNCCATACCTAGTATAAACAATGAGACA |
| **F3_A8_R** | CTGAACCGCTCTTCCGATCTNNNNNNTAAGTCAGATCCTACATACAAATCATC |
| **F3_A9_FK** | CACGACGCTCTTCCGATCTKKNNNNCCAGACATAGTCATCTATCAATACATG |
| **F3_A9_FM** | CACGACGCTCTTCCGATCTMMNNNNCCAGACATAGTCATCTATCAATACATG |
| **F3_A9_R** | CTGAACCGCTCTTCCGATCTNNNNNNTTATCAGGATGGAGTTCATAACC |
| **F3_A10_FK** | CACGACGCTCTTCCGATCTKKNNNNGAACCTCCATTCCTTTGGATG |
| **F3_A10_FM** | CACGACGCTCTTCCGATCTMMNNNNGAACCTCCATTCCTTTGGATG |
| **F3_A10_R** | CTGAACCGCTCTTCCGATCTNNNNNNGTTCCCCTAAGAAGTTTACATAATTG |
| **F3_A11_FK** | CACGACGCTCTTCCGATCTKKNNNNATGACATACAGAAATTAGTGGGAA |
| **F3_A11_FM** | CACGACGCTCTTCCGATCTMMNNNNATGACATACAGAAATTAGTGGGAA |
| **F3_A11_R** | CTGAACCGCTCTTCCGATCTNNNNNNCACTCCATGTACCGGTTCTTTTAG |
| **F4_A1_FK** | CACGACGCTCTTCCGATCTKKNNNNCTAAAAGAACCGGTACATGGAGTG |
| **F4_A1_FM** | CACGACGCTCTTCCGATCTMMNNNNCTAAAAGAACCGGTACATGGAGTG |
| **F4_A1_R** | CTGAACCGCTCTTCCGATCTNNNNNNACTGCCTCTGTTAATTGTTTCAC |
| **F4_A2_FK** | CACGACGCTCTTCCGATCTKKNNNNGGGTGCCCACACTAATGAT |
| **F4_A2_FM** | CACGACGCTCTTCCGATCTMMNNNNGGGTGCCCACACTAATGAT |
| **F4_A2_R** | CTGAACCGCTCTTCCGATCTNNNNNNGAGGGGTATTGACAAACTCCC |
| **F4_A3_FK** | CACGACGCTCTTCCGATCTKKNNNNCCACCTGGATTCCTGAGT |
| **F4_A3_FM** | CACGACGCTCTTCCGATCTMMNNNNCCACCTGGATTCCTGAGT |
| **F4_A3_R** | CTGAACCGCTCTTCCGATCTNNNNNNCGTTAGGGGGACAACTTTTTG |
| **F4_A4_FK** | CACGACGCTCTTCCGATCTKKNNNNGGATATGTAACTGACAGAGGAAGA |
| **F4_A4_FM** | CACGACGCTCTTCCGATCTMMNNNNGGATATGTAACTGACAGAGGAAGA |
| **F4_A4_R** | CTGAACCGCTCTTCCGATCTNNNNNNACTAACTCTGATTCACTCTTATCTGG |
| **F4_A5_FK** | CACGACGCTCTTCCGATCTKKNNNNCATTGGGAATCATTCAAGCACAA |
| **F4_A5_FM** | CACGACGCTCTTCCGATCTMMNNNNCATTGGGAATCATTCAAGCACAA |
| **F4_A5_R** | CTGAACCGCTCTTCCGATCTNNNNNNTCCATCTAAAAATAGTACTTTCCTGATTC |
| **F4_A6_FK** | CACGACGCTCTTCCGATCTKKNNNNATGGGTTGGTCAGTGCTG |
| **F4_A6_FM** | CACGACGCTCTTCCGATCTMMNNNNATGGGTTGGTCAGTGCTG |
| **F4_A6_R** | CTGAACCGCTCTTCCGATCTNNNNNNATGCATGGCTTCCCCTT |
| **F4_A7_FK** | CACGACGCTCTTCCGATCTKKNNNNAGTAGCCAGCTGTGATAAATGTC |
| **F4_A7_FM** | CACGACGCTCTTCCGATCTMMNNNNAGTAGCCAGCTGTGATAAATGTC |
| **F4_A7_R** | CTGAACCGCTCTTCCGATCTNNNNNNATGCTGTTTCTTGCCCTGT |
| **F4_A8_FK** | CACGACGCTCTTCCGATCTKKNNNNGCAGAAGTAATTCCAGCAGAG |
| **F4_A8_FM** | CACGACGCTCTTCCGATCTMMNNNNGCAGAAGTAATTCCAGCAGAG |
| **F4_A8_R** | CTGAACCGCTCTTCCGATCTNNNNNNTGACTTTGGGGATTGTAGGG |
| **F4_A9_FK** | CACGACGCTCTTCCGATCTKKNNNNGATCAAGCAGGAATTTGGCA |
| **F4_A9_FM** | CACGACGCTCTTCCGATCTMMNNNNGATCAAGCAGGAATTTGGCA |
| **F4_A9_R** | CTGAACCGCTCTTCCGATCTNNNNNNCTGCACTGTACCCCCCAA |
| **F4_A10_FK** | CACGACGCTCTTCCGATCTKKNNNNCAAATGGCAGTATTCATCCACAA |
| **F4_A10_FM** | CACGACGCTCTTCCGATCTMMNNNNCAAATGGCAGTATTCATCCACAA |
| **F4_A10_R** | CTGAACCGCTCTTCCGATCTNNNNNNGCTGGTCCTTTCCAAACTG |
| **F4_A11_FK** | CACGACGCTCTTCCGATCTKKNNNNTACAGGGACAGCAGAGATC |
| **F4_A11_FM** | CACGACGCTCTTCCGATCTMMNNNNTACAGGGACAGCAGAGATC |
| **F4_A11_R** | CTGAACCGCTCTTCCGATCTNNNNNNTAATCCTCATCCTGTCTACTTGC |
| **F4_A12_FK** | CACGACGCTCTTCCGATCTKKNNNNGGCAGGTGATGATTGTGTG |
| **F4_A12_FM** | CACGACGCTCTTCCGATCTMMNNNNGGCAGGTGATGATTGTGTG |
| **F4_A12_R** | CTGAACCGCTCTTCCGATCTNNNNNNTTAGCATCCCCTAGTGGGAT |
| **F4_A13_FK** | CACGACGCTCTTCCGATCTKKNNNNATCCAAAAATAAGTTCAGAAGTACAC |
| **F4_A13_FM** | CACGACGCTCTTCCGATCTMMNNNNATCCAAAAATAAGTTCAGAAGTACAC |
| **F4_A13_R** | CTGAACCGCTCTTCCGATCTNNNNNNGTGCAGATGAATTAGTTGGTCTG |
| **F4_A14_FK** | CACGACGCTCTTCCGATCTKKNNNNCACAAGTAGACCCTGACCTAG |
| **F4_A14_FM** | CACGACGCTCTTCCGATCTMMNNNNCACAAGTAGACCCTGACCTAG |
| **F4_A14_R** | CTGAACCGCTCTTCCGATCTNNNNNNTGCTGCTAGTGCCAAGTAC |
| **F4_A15_FK** | CACGACGCTCTTCCGATCTKKNNNNGCAGGACATAACAAGGTAGGA |
| **F4_A15_FM** | CACGACGCTCTTCCGATCTMMNNNNGCAGGACATAACAAGGTAGGA |
| **F4_A15_R** | CTGAACCGCTCTTCCGATCTNNNNNNAAGTTCCTCTAAAAGCTCTAGTGTC |
| **F4_A16_FK** | CACGACGCTCTTCCGATCTKKNNNNAGAAGACCAAGGGCCACA |
| **F4_A16_FM** | CACGACGCTCTTCCGATCTMMNNNNAGAAGACCAAGGGCCACA |
| **F4_A16_R** | CTGAACCGCTCTTCCGATCTNNNNNNGTTGTTGCAGAATTCTTATTATGGC |
| **F5_A1_FK** | CACGACGCTCTTCCGATCTKKNNNNCATAATAAGAATTCTGCAACAACTGC |
| **F5_A1_FM** | CACGACGCTCTTCCGATCTMMNNNNCATAATAAGAATTCTGCAACAACTGC |
| **F5_A1_R** | CTGAACCGCTCTTCCGATCTNNNNNNGCAACACTTTTTACAATAGCAATTGG |
| **F5_A2_FK** | CACGACGCTCTTCCGATCTKKNNNNGAAGTCAGCCTAAAACTGCTTG |
| **F5_A2_FM** | CACGACGCTCTTCCGATCTMMNNNNGAAGTCAGCCTAAAACTGCTTG |
| **F5_A2_R** | CTGAACCGCTCTTCCGATCTNNNNNNCATGTACTACTTACTGCTTTGATAGAG |
| **F5_A3_FK** | CACGACGCTCTTCCGATCTKKNNNNGAACAGTCAGACTCATCAAGC |
| **F5_A3_FM** | CACGACGCTCTTCCGATCTMMNNNNGAACAGTCAGACTCATCAAGC |
| **F5_A3_R** | CTGAACCGCTCTTCCGATCTNNNNNNAGTCTATCAATTAACCTGTCTATTTTTC |
| **F5_A4_FK** | CACGACGCTCTTCCGATCTKKNNNNGTTGTGTGGTCCATAGTAATCATAG |
| **F5_A4_FM** | CACGACGCTCTTCCGATCTMMNNNNGTTGTGTGGTCCATAGTAATCATAG |
| **F5_A4_R** | CTGAACCGCTCTTCCGATCTNNNNNNTACAGATCATCAATATCCCAAGGAG |
| **F5_A5_FK** | CACGACGCTCTTCCGATCTKKNNNNGGAAATGGGGCACCATG |
| **F5_A5_FM** | CACGACGCTCTTCCGATCTMMNNNNGGAAATGGGGCACCATG |
| **F5_A5_R** | CTGAACCGCTCTTCCGATCTNNNNNNTGTGGGTACACAGGCATG |
| **F5_A6_FK** | CACGACGCTCTTCCGATCTKKNNNNGGTACATAATGTTTGGGCCAC |
| **F5_A6_FM** | CACGACGCTCTTCCGATCTMMNNNNGGTACATAATGTTTGGGCCAC |
| **F5_A6_R** | CTGAACCGCTCTTCCGATCTNNNNNNCACAGAGTGGGGTTAATTTTACAC |
| **F5_A7_FK** | CACGACGCTCTTCCGATCTKKNNNNGGGATCAAAGCCTAAAGCC |
| **F5_A7_FM** | CACGACGCTCTTCCGATCTMMNNNNGGGATCAAAGCCTAAAGCC |
| **F5_A7_R** | CTGAACCGCTCTTCCGATCTNNNNNNTGCATATTCTTTCTGCACCTTATC |
| **F5_A8_FK** | CACGACGCTCTTCCGATCTKKNNNNGCTCTTTCAATATCAGCACAAGC |
| **F5_A8_FM** | CACGACGCTCTTCCGATCTMMNNNNGCTCTTTCAATATCAGCACAAGC |
| **F5_A8_R** | CTGAACCGCTCTTCCGATCTNNNNNNAGCCGGGGCACAATAATGTAT |
| **F5_A9_FK** | CACGACGCTCTTCCGATCTKKNNNNGTATCCTTTGAGCCAATTCCC |
| **F5_A9_FM** | CACGACGCTCTTCCGATCTMMNNNNGTATCCTTTGAGCCAATTCCC |
| **F5_A9_R** | CTGAACCGCTCTTCCGATCTNNNNNNACATCTTCTTCTGCTAGACTGC |
| **F5_A10_FK** | CACGACGCTCTTCCGATCTKKNNNNGTATCAACTCAACTGCTGTTAAATG |
| **F5_A10_FM** | CACGACGCTCTTCCGATCTMMNNNNGTATCAACTCAACTGCTGTTAAATG |
| **F5_A10_R** | CTGAACCGCTCTTCCGATCTNNNNNN GCT CTC CCT GGT CCC CT |
| **F5_A11_FK** | CACGACGCTCTTCCGATCTKKNNNNTCTGTAGAAATTAATTGTACAAGACCC |
| **F5_A11_FM** | CACGACGCTCTTCCGATCTMMNNNNTCTGTAGAAATTAATTGTACAAGACCC |
| **F5_A11_R** | CTGAACCGCTCTTCCGATCTNNNNNNGTTCTCTTAATTTGCTAGCTATCTG |
| **F6_A1_FK** | CACGACGCTCTTCCGATCTKKNNNNCAGATAGCTAGCAAATTAAGAGAAC |
| **F6_A1_FM** | CACGACGCTCTTCCGATCTMMNNNNCAGATAGCTAGCAAATTAAGAGAAC |
| **F6_A1_R** | CTGAACCGCTCTTCCGATCTNNNNNNTGACCCTTCAGTACTCCAAG |
| **F6_A2_FK** | CACGACGCTCTTCCGATCTKKNNNNCAACACAACTGTTTAATAGTACTTGG |
| **F6_A2_FM** | CACGACGCTCTTCCGATCTMMNNNNCAACACAACTGTTTAATAGTACTTGG |
| **F6_A2_R** | CTGAACCGCTCTTCCGATCTNNNNNNGAACATCTAATTTGTCCACTGATGG |
| **F6_A3_FK** | CACGACGCTCTTCCGATCTKKNNNNAAGCAATGTATGCCCCTC |
| **F6_A3_FM** | CACGACGCTCTTCCGATCTMMNNNNAAGCAATGTATGCCCCTC |
| **F6_A3_R** | CTGAACCGCTCTTCCGATCTNNNNNNTTCACTTCTCCAATTGTCCC |
| **F6_A4_FK** | CACGACGCTCTTCCGATCTKKNNNNACCTGGAGGAGGCGATATG |
| **F6_A4_FM** | CACGACGCTCTTCCGATCTMMNNNNACCTGGAGGAGGCGATATG |
| **F6_A4_R** | CTGAACCGCTCTTCCGATCTNNNNNNCGCCCATAGTGCTTCCTG |
| **F6_A5_FK** | CACGACGCTCTTCCGATCTKKNNNNTTGGGTTCTTGGGAGCAG |
| **F6_A5_FM** | CACGACGCTCTTCCGATCTMMNNNNTTGGGTTCTTGGGAGCAG |
| **F6_A5_R** | CTGAACCGCTCTTCCGATCTNNNNNNAGCCAGGATTCTTGCCTGG |
| **F6_A6_FK** | CACGACGCTCTTCCGATCTKKNNNNCTGGGGCATCAAACAGCT |
| **F6_A6_FM** | CACGACGCTCTTCCGATCTMMNNNNCTGGGGCATCAAACAGCT |
| **F6_A6_R** | CTGAACCGCTCTTCCGATCTNNNNNNTGTCCCACTCCATCCAGG |
| **F6_A7_FK** | CACGACGCTCTTCCGATCTKKNNNNCTGGAACAGATTTGGAATAACATGA |
| **F6_A7_FM** | CACGACGCTCTTCCGATCTMMNNNNCTGGAACAGATTTGGAATAACATGA |
| **F6_A7_R** | CTGAACCGCTCTTCCGATCTNNNNNNTACCACAGCCAATTTGTTATGTTA |
| **F6_A8_FK** | CACGACGCTCTTCCGATCTKKNNNNGGGCAAGTTTGTGGAATTGG |
| **F6_A8_FM** | CACGACGCTCTTCCGATCTMMNNNNGGGCAAGTTTGTGGAATTGG |
| **F6_A8_R** | CTGAACCGCTCTTCCGATCTNNNNNNTCCCCTCGGGATTGGGA |
| **F6_A9_FK** | CACGACGCTCTTCCGATCTKKNNNNCATTATCGTTTCAGACCCACC |
| **F6_A9_FM** | CACGACGCTCTTCCGATCTMMNNNNCATTATCGTTTCAGACCCACC |
| **F6_A9_R** | CTGAACCGCTCTTCCGATCTNNNNNNCAAGAGTAAGTCTCTCAAGCGG |
| **F6_A10_FK** | CACGACGCTCTTCCGATCTKKNNNNGTGCCTCTTCAGCTACCA |
| **F6_A10_FM** | CACGACGCTCTTCCGATCTMMNNNNGTGCCTCTTCAGCTACCA |
| **F6_A10_R** | CTGAACCGCTCTTCCGATCTNNNNNNTCCCCTCAGCTACTGCTATG |
| **F6_A11_FK** | CACGACGCTCTTCCGATCTKKNNNNCTTGCTCAATGCCACAGC |
| **F6_A11_FM** | CACGACGCTCTTCCGATCTMMNNNNCTTGCTCAATGCCACAGC |
| **F6_A11_R** | CTGAACCGCTCTTCCGATCTNNNNNNCCCTTACAGCAGGCCAT |
| **F6_A12_FK** | CACGACGCTCTTCCGATCTKKNNNNGCAGCTTATAGAGCTATTCGC |
| **F6_A12_FM** | CACGACGCTCTTCCGATCTMMNNNNGCAGCTTATAGAGCTATTCGC |
| **F6_A12_R** | CTGAACCGCTCTTCCGATCTNNNNNNCTAGGTCTCGAGATACTGCTC |
| **F7_A1_FK** | CACGACGCTCTTCCGATCTKKNNNNGAGCAGTATCTCGAGACCTAG |
| **F7_A1_FM** | CACGACGCTCTTCCGATCTMMNNNNGAGCAGTATCTCGAGACCTAG |
| **F7_A1_R** | CTGAACCGCTCTTCCGATCTNNNNNNAGTGGCTAAGATCTACAGCTG |
| **F7_A2_FK** | CACGACGCTCTTCCGATCTKKNNNNCCTTTAAGACCAATGACTTACAAGG |
| **F7_A2_FM** | CACGACGCTCTTCCGATCTMMNNNNCCTTTAAGACCAATGACTTACAAGG |
| **F7_A2_R** | CTGAACCGCTCTTCCGATCTNNNNNNAGTGGATATCTGACCCCTG |
| **F7_A3_FK** | CACGACGCTCTTCCGATCTKKNNNNGAACTACACACCAGGGC |
| **F7_A3_FM** | CACGACGCTCTTCCGATCTMMNNNNGAACTACACACCAGGGC |
| **F7_A3_R** | CTGAACCGCTCTTCCGATCTNNNNNNGGCTGTCAAACCTCCAC |
| **F7_A4_FK** | CACGACGCTCTTCCGATCTKKNNNNGACCCTGAGAGAGAAGTGTTAGA |
| **F7_A4_FM** | CACGACGCTCTTCCGATCTMMNNNNGACCCTGAGAGAGAAGTGTTAGA |
| **F7_A4_R** | CTGAACCGCTCTTCCGATCTNNNNNNCATCTGAGGGCTCGCCA |
| **F7_A5_FK** | CACGACGCTCTTCCGATCTKKNNNNTGGCCTGGGCGGGACT |
| **F7_A5_FM** | CACGACGCTCTTCCGATCTMMNNNNTGGCCTGGGCGGGACT |
| **F7_A5_R** | CTGAACCGCTCTTCCGATCTNNNNNNCTTGAAGCACTCAAGGCAAGCT |
| **NGS preparation PCR 2: Illumina specific** | |
| **5’-Univ_FC** | AATGATACGGCGACCACCGAGATCTACACTCTTTCCCTACACGACGCTCTTCCG |
| **3’-Univ_FC** | CAAGCAGAAGACGGCATACGAGATCGGTCTCGGCATTCCTGCTGAACCGCTCTTCCG |
| **HIV-1 individual mutant construction** | |
| **CA A194T-F** | CAGAAACCTTGTTGGTCCAAAATACGAACCCAGATTGTAAGACTA |
| **CA A194T-R** | TAGTCTTACAATCTGGGTTCGTATTTTGGACCAACAAGGTTTCTG |
| **PR D25G-F** | GGGCAATTAAAGGAAGCTCTATTAGGTACAGGAGCAGATGATACAGTATTAG |
| **PR D25G-R** | CTAATACTGTATCATCTGCTCCTGTACCTAATAGAGCTTCCTTTAATTGCCC |
| **PR D29G-F** | GGAAGCTCTATTAGATACAGGAGCAGGTGATACAGTATTAGAAGAAATGAATTTGCC |
| **PR D29G-R** | GGCAAATTCATTTCTTCTAATACTGTATCACCTGCTCCTGTATCTAATAGAGCTTCC |
| **RT E6K-F** | GCACTTTAAATTTTCCCATTAGTCCTATTAAGACTGTACCAGTAAAATTAAAGCCAGG |
| **RT E6K-R** | CCTGGCTTTAATTTTACTGGTACAGTCTTAATAGGACTAATGGGAAAATTTAAAGTGC |
| **RT F61S-F** | CTGAAAATCCATACAATACTCCAGTATCTGCCATAAAGAAAAAAGACAGTACTAAATG |
| **RT F61S-R** | CATTTAGTACTGTCTTTTTTCTTTATGGCAGATACTGGAGTATTGTATGGATTTTCAG |
| **vif D101N-F** | GATATAGCACACAAGTAGACCCTAACCTAGCAGACCAACTAATTCATCTG |
| **vif D101N-R** | CAGATGAATTAGTTGGTCTGCTAGGTTAGGGTCTACTTGTGTGCTATATC |
| **RT Y501C-F** | GAAGTAAACATAGTGACAGACTCACAATGTGCATTGGGAATCATTCAAGCAC |
| **RT Y501C-R** | GTGCTTGAATGATTCCCAATGCACATTGTGAGTCTGTCACTATGTTTACTTC |
| **IN N155Y-F** | CAAAGTCAAGGAGTAATAGAATCTATGTATAAAGAAT TAAAGA AAATTATAGGACAGGT |
| **IN N155Y-R** | ACCTGTCCTATAATTTTCTTTAATTCTTTATACATAGATTCTATTACTCCTTGACTTTG |
| **rev E10G-F** | GCGGAGACAGCGACGGAGAGCTCATCAGAACAGTCAG AC |
| **rev E10G-R** | GTCTGACTGTTCTGATGAGCTCTCCGTCGCTGTCTCCGC |
| **gp120 C119G-F** | GGGATCAAAGCCTAAAGCCAGGTGTAA AATTAACCCCACTCTGTGT |
| **gp120 C119G-R** | ACACAGAGTGGGGTTAATTTTACACCTGGCTTTAGGCTTTGATCCC |
| **gp120 K205M-F** | CATTACACAGGCCTGTCCAATGGTATCCTTTGAGCCAATTCCC |
| **gp120 K205M-R** | GGGAATTGGCTCAAAGGATACCATTGGACAGGCCTGTGTAATG |
| **gp120 D476V-F** | AGACCTGGAGGAGGCGTTATGAGGGACAATTGGAGAAGTG |
| **gp120 D476V-R** | CACTTCTCCAATTGTCCCTCATAACGCCTCCTCCAGGTCT |
| **gp41 Y136H-F** | GGAGTGGGACAGAGAAAT TAACAATCACACAAGCTTAATACACTCCTTAATTGAAG |
| **gp41 Y136H-R** | CTTCAATTAAGGAGTGTATTAAGCTTGTGTGATTGTTAATTTCTCTGTCCCACTCC |
| **3'LTR C9547T-F** | GTACTGGGTCTCTCTGGTTAGACTAGATCTGAGCCTGGGAGC |
| **3'LTR C9547T-R** | GCTCCCAGGCTCAGATCTAGTCTAACCAGAGAGACCCAGTAC |
